# Identification and Molecular Characterization of Two Acetylcholinesterases from the Salmon Louse, *Lepeophtheirus salmonis*

**DOI:** 10.1371/journal.pone.0125362

**Published:** 2015-05-04

**Authors:** Kiranpreet Kaur, Marit Jørgensen Bakke, Frank Nilsen, Tor Einar Horsberg

**Affiliations:** 1 NMBU School of Veterinary Science, Sea Lice Research Centre, PO Box 8146 Dep., NO-0033 Oslo, Norway; 2 University of Bergen, Department of Biology, Sea Lice Research Centre, NO-5020 Bergen, Norway; Weizmann Institute of Science, ISRAEL

## Abstract

Acetylcholinesterase (AChE) is an important enzyme in cholinergic synapses. Most arthropods have two genes (*ace1* and *ace2*), but only one encodes the predominant synaptic AChE, the main target for organophosphates. Resistance towards organophosphates is widespread in the marine arthropod *Lepeophtheirus salmonis*. To understand this trait, it is essential to characterize the gene(s) coding for AChE(s). The full length cDNA sequences encoding two AChEs in *L*. *salmonis* were molecularly characterized in this study. The two ace genes were highly similar (83.5% similarity at protein level). Alignment to the *L*. *salmonis* genome revealed that both genes were located close to each other (separated by just 26.4 kbp on the *L*. *salmonis* genome), resulting from a recent gene duplication. Both proteins had all the typical features of functional AChE and clustered together with AChE-type 1 proteins in other species, an observation that has not been described in other arthropods. We therefore concluded the presence of two versions of *ace1* gene in *L*. *salmonis*, named *ace1a* and *ace1b*. *Ace1a* was predominantly expressed in different developmental stages compared to *ace1b* and was possibly active in the cephalothorax, indicating that *ace1a* is more likely to play the major role in cholinergic synaptic transmission. The study is essential to understand the role of AChEs in resistance against organophosphates in *L*. *salmonis*.

## Introduction

Acetylcholinesterase (AChE) is a serine hydrolase whose primary function is to terminate synaptic transmission at cholinergic synapses and neuromuscular junctions of both vertebrates and invertebrates by hydrolyzing the neurotransmitter acetylcholine (ACh) [1]. AChE has been studied extensively in relation to resistance against two main classes of anti-cholinergic agents, organophosphates (OP) and carbamates (CB). AChE is the primary target site for these chemicals, which react covalently with the active site serine of the enzyme, a part of the catalytic triad [2]. The binding blocks the cleavage of the transmitter ACh and results in elevated levels of ACh in the synaptic cleft, thereby causing excitation, paralysis and death of the organism. The biochemical and molecular characterization of AChE genes has been reported mostly in insects such as *Drosophila melanogaster* [3], *Anopheles stephensi* [4], *Leptinotarsa decemlineata* [5] *Musca domestica* [6], *Schizaphis graminum* [7], *Blattella germanica* [8] and *Cimex lectularius* [9].

In contrast to vertebrates, different forms of AChE are encoded by separate genes in invertebrates. For example, most of the arthropods have two AChE genes (*ace1* and *ace2*), of which only one (*ace1*) usually encodes the predominant synaptic AChE (AChE1) and is involved in OP and CB insensitivity mechanisms [8 – 11]. Similar to arthropods, acarids have also been reported to have two AChE genes. For example, *Pardosa pseudoannulata* has two AChE genes (*ace1* and *ace2*), of which *ace1* is the major synaptic enzyme [12]. Whereas three genes coding for AChE were identified in *Rhipicephalus microplus*; these three AChE genes exhibit different amino acid sequences and biochemical properties. However, both ace1 and ace2 are apparently expressed in synganglia and harbor OP resistance associated mutations [13].

On the contrary, only the *ace2* locus is present in Cyclorrapha dipterans (*D*. *melanogaster*, *Lucilia cuprina*, *M*. *domestica* and *Bactrocera oleae*), coding for a single functional AChE (AChE2) involved in synaptic transmission and resistance mechanisms against OPs and CBs [14–17]. However, only *ace1* codes for a functional AChE and plays a role in insensitivity mechanisms in most of the non-Cyclorrapha arthropods. Nematodes, on the other hand, (*Caenorhabditis elegans*) have four genes (*ace1*, *ace2*, *ace3* and *ace4*) for AChEs, of which three code for functional enzymes [18].

Generally, *ace1* has been reported to be the main AChE in the synaptic transmission in cholinergic synapses [8–11]. However, according to a study carried out by Kim and Lee, 2013, a*ce2* was observed as the major catalytic enzyme in 33 out of 100 insect species studied [19].

Duplication of AChE is also associated with OP resistance in fruit flies, mosquitoes and acarids. For example, in *Drosophila melanogaster* the amount of AChE is directly correlated with insecticide resistance [20]. In *Culex pipiens*, duplication of AChE in response to insecticide pressure has been observed [21] and resistant strains of *Tetranychus urticae* have also been shown to possess more copies of AChE than the sensitive strains [22].

*Lepeophtherius salmonis* (Copepoda: Caligidae) commonly referred to as the salmon louse, occasionally also sea louse, is an ecto-parasitic copepod infesting different salmonid species. They feed on mucus, epidermal tissue and blood of salmonid fish in sea water. Chemical controls using OPs from the late 1970s until the mid-1990’s have been the major approach in Norway to control *L*. *salmonis* infestations on farmed salmonids. Since 2008, the use of OPs in Norwegian aquaculture has again been increased. Similar to other arthropods, the frequent use of OP over the years resulted in the development of resistance in *L*. *salmonis* against them in the 1990s. This has resulted in economic loss afflicting the aquaculture industry [23].

Understanding and unravelling biochemical pathways underlying the resistance in *L*. *salmonis* against OP is the need of the hour. However, in order to understand these biochemical pathways, it is essential to characterize the gene(s) coding for AChE in *L*. *salmonis* and to determine whether AChE1 or AChE2 is responsible for insensitivity against OPs. Unfortunately, no study on the characterization of AChE(s) in *L*. *salmonis* is available in the present literature. Hence, we aimed to identify and characterize the gene(s) coding for AChE(s) in *L*. *salmonis* in this study.

## Materials and Methods

### Samples of salmon lice

Salmon lice samples from a strain (Ls A) with no history of insensitivity against azamethiphos (as per bioassay results) were collected from newly slaughtered fish at a commercial fish processing plant in 2010. The fish and the parasites originated from the western part of the county Finnmark in Northern Norway. Parasites were subsequently cultivated for approx. 10 generations on Atlantic salmon in the laboratory at the NIVA Marine Research Station at Solbergstrand, Drøbak, Norway. Samples from these fish were collected after anesthesia of the fish with metacaine (125 mg/L for 2 minutes).

### Total RNA extraction and cDNA synthesis

Total RNA was extracted using RNeasy plus Mini kit (Qiagen, CA, USA), from female adult individuals, as per manufacturer’s protocol. The RNA was quantified and qualified on ND-100 Spectrophotometer (Thermo Fisher Scientific, DE, USA). First strand cDNA was synthesized from total RNA (1 μg) using qScript reverse transcriptase (Quanta Biosciences, MD, USA).

### Partial cDNA fragments of AChE genes

Conserved cDNA sequences of AChEs were selected from other species using the GenBank database. These selected sequences were then compared against the salmon louse genome database (Viroblast; sealouse.imr.no) to obtain the homologous sequences in salmon lice, which were then amplified using specific primers followed by direct sequencing. The sequences obtained (after direct sequencing) were again compared against the salmon lice genome using homology blast in order to confirm that only two matches (referred to as *ace1a* and *ace1b* hereafter) for AChEs existed in the *L*. *salmonis* genome.

### Rapid amplification of cDNA ends

5’ and 3’ ends of partial cDNAs, obtained by homology blast, were amplified using Rapid amplification of cDNA ends (RACE) with sequence specific primers (listed in S1 File) and SMART RACE kit (Clontech, Palo Alto, CA, USA) as per manufacturer’s instructions. RACE PCR conditions: 5 cycles at 94^°^C for 30 s, 72^°^C for 3 min followed by 5 cycles at 94^°^C for 30 s, 70^°^C for 30 s, 72^°^C for 3 min followed by 25 cycles at 94^°^C for 30 s, 68^°^C for 30 s and 72^°^C for 3 min.

Both 5’RACE and 3’RACE PCR products were cloned using TOPO TA Cloning Kit for sequencing (Invitrogen, CA, USA) followed by isolation of plasmid DNA from positive colonies using PureLink Quick Plasmid Miniprep kit (Invitrogen, CA, USA) under manufacturer’s instructions. Amplicons were obtained using the plasmid DNA and TOPO vector specific primers (listed in S1 File) under PCR conditions: 94^°^C for 4 min followed by 30 cycles at 94^°^C for 1 min, 52^°^C for 1 min, 72^°^C for 1 min and followed by final extension at 72^°^C for 5 min. Amplicons were then subjected to direct sequencing using BIG Dye Terminator v3.1 cycle sequencing kit (Life technologies, Invitrogen, CA, USA) on a 3130xl Genetic Analyzer (ABI Prism, Life technologies, Invitrogen, CA, USA) to obtain the full length sequence of cDNAs.

### Comparison of *L*. *salmonis* AChEs with other species

Deduced amino acid sequences of AChE1a and AChE1b were compared with previously published AChE protein sequences from other species, using ClustalW program with BLOSUM matrix and default settings to obtain Multiple sequence alignment (MSA).

### Phylogenetic analysis

Phylogenetic analysis was performed on the Phylogeney.fr platform (http://www.phylogeny.fr/version2_cgi/index.cgi), [24]. The amino acid sequences were aligned with MUSCLE (v3.7) [25] and configured for highest accuracy. After alignment, the phylogeney.fr platform removed ambiguous regions (containing gaps and/or poorly aligned residues) with Gblocks (v0.91b), and constructed the phylogenetic tree using the maximum likelihood method implemented in the PhyML program (v3.0 aLRT) [26]. The WAG substitution model was selected assuming an estimated proportion of invariant sites (of 0.105) and 4 gamma-distributed rate categories to account for the rate heterogeneity across sites. Reliability for internal branch was assessed using bootstrapping method (100 bootstrap replicates).

### Genomic organization of *L*. *salmonis* AChEs

The complete cDNA sequences for *ace1a* and *ace1b* obtained after RACE PCR were used to blast against the *L*. *salmonis* assembly (sealouse.imr.no) in order to find the organization of the two genes in the *L*. *salmonis* genome. This assembly will be made publically available in 2015.

### Quantitative analysis of *L*. *salmonis* ace1a and ace1b

Total RNA was extracted from 6 different (nauplius I, nauplius II, copepodid, chalimus, preadult and adult) developmental stages of *L*. *salmonis* using RNeasy plus Mini kit (Qiagen, CA, USA), as mentioned above. First strand cDNA was synthesized from total RNA (1 μg) using qScript reverse transcriptase (Quanta Biosciences, MD, USA) and 100 ng was subsequently used as PCR template for qPCR using gene specific primers (listed in S1 File) and SsoAdvanced SYBR Green supermix (New England BioLabs, MA, USA), as per manufacturer’s protocol. The Elongation Factor (EF) gene was used as an internal standard. After qPCR, the homogeneity of PCR products was confirmed by melting curve analysis. The range of acceptable efficiencies for qPCR analysis was 0.90–1.0. Relative transcription levels were determined by the following equation:

$$\text{ratio}=2^{-(\text{Ct}}{{}^{\text{target}}}^{-\text{Ct}}{{}^{\text{reference}}}^{)}$$

From this equation, relative transcription levels of *ace1a* and *ace1b* were calculated by equations:

$$\text{ratio}=2^{\text{-(Ct}}{{}^{\text{ace1a}}}^{-\text{Ct}}{{}^{\text{EF}}}^{)}{\text{and}2}^{\text{-}(\text{Ct}}{{}^{\text{ace1b}}}^{-\text{Ct}}{{}^{\text{EF}}}^{)}$$

Two separate samples were run in duplicate and used for calculation of the mean and STD. Fold different change between *ace1a* and *ace1b* was calculated according to the 2^-Δ(ΔCt)^ method [27]. Two negative controls were added to each reaction, a non-template control and a non-amplicon control (-RT control).

### Protein sample preparation, Native PAGE, activity staining

The cephalothorax and the posterior tissues were separated as close to the cephalothorax segment as possible from female adult salmon louse using a sterile scalpel. Proteins were extracted from the cephalothorax, the posterior tissues and the whole body tissues of female adult lice. Briefly, the membrane bound proteins were extracted with 0.1M Tris-HCl buffer supplemented with 0.5% Triton X-100, using a tissue homogenizer. The homogenates were then centrifuged at 12,000 g for 15 minutes at 4^°^C. The supernatant was stored at -80^°^C until further use.

The Native PAGE was performed as per Kim *et al*. 2012 [28] in a vertical electrophoresis unit (Novex mini cell, Invitrogen, CA, USA). Protein preparations were loaded in triplicates (50 μg) in 8% native PAGE gel (Invitrogen, CA, USA) and separated at 120V for 100 min in a cold chamber (4^°^C) with continuous Tris-glycine buffer system supplemented with 0.5% Triton X-100. Following electrophoresis, one set of gel was used for AChE activity staining and bands were visualized according to Lewis and Shute [29]. The other two set of gels were used for Western blot analysis (listed in S2 File).

Molecular mass and isoelectric points were predicted by compute pI/Mw tool (http:us.expasy.org/tools/pi_tool.html).

### 3D modelling of the enzymes

The three-dimensional structure of the enzyme was modelled using SWISS MODEL in the automated mode [30], http://swissmodel.expasy.org/. An initial template search revealed several possible templates. The eight highest ranked templates were evaluated using the root mean square function (RSM) for the fit between the template and the *L*. *salmonis* AChE1a structure predicted from the template. The best fit was found for native AChE from *D*. *melanogaster* as template (RSM 0.25 for the whole protein, 0.05 for the ten amino acids important for choline binding, the catalytic triad, the acyl pocket and the oxyanion hole). The other templates gave RSMs of 1.39–4.43 for the whole protein and 0.13–9.04 for the ten essential amino acids, respectively. The template producing the best fit was generated on basis of the crystalline structure of the *D*. *melanogaster* AChE protein, determined by X-ray diffraction [31], Protein Data Bank (PDB) code 1QO9. This template also produced a good fit for *L*. *salmonis* AChE1b (RMS 0.27 for the whole protein, 0.05 for the ten important amino acids). The numbering of amino acids is by convention from the *Torpedo californica* protein sequence.

### In situ hybridization

The parasites used for *in situ* hybridization were collected alive and immersed in 4% buffered paraformaldehyde (PFA) under RNAse free conditions and with RNAse free PBS. An automated system was used for the exchange of PFA to paraffin before embedding. Sequential sections (3 μm) of parasites at different developmental stages were collected on SuperFrost Plus (Thermo Scientific) slides. The slides were kept in RNAse free boxes until further processing.

Antisense and sense locked nucleic acid (LNA) probes (listed in S1 File), labeled with digoxiginin (DIG) in both 3’ and 5’ ends, were ordered for *ace1a* and *ace1b* (Exiqon A/S, Denmark). There was a 7 nucleotide difference between the two probes. The designed probes were blasted against the *L*. *salmonis* genome assembly (Viroblast; sealouse.imr.no) to check for possible cross reactions. A difference of 4–5 nucleotides between the target gene and other genes was considered to be a sufficient difference. Unfortunately, the probe towards *ace1b* was not specific for this gene and a second blast identified a similarity with RNA coding for ribosomal proteins. Since this protein is present in all cells in rather large quantities, positive staining occurred, although the probe lacked a perfect match. Thus, the probe most likely bound weakly to RNA for ribosomal proteins in all cells, and could only be used as a positive control.

*In situ* hybridization was carried out using a combination of previously described protocols [32–34] on copepodids (gender unknown), preadult II (female) and adult (female) lice. The samples were deparaffinized (baking at 60 °C for 30 minutes), rinsed three times for 10 minutes (Histoclear, National Diagnostics, Hull, England) and rehydrated (ethanol at 2x100%, 1x95%, 1x70% and 1x50%, 1 min intervals). After a short wash (1 min) in phosphate-buffered saline (PBS), fixation in cold PFA (buffered, 4%, 5 minutes), and another washing in PBS (2X3 minutes), the probes were acetylated with 0.25% acetic anhydride in triethanolamine (10 minutes) followed by Protein K digestion with a 5 minutes wash (PBS) in between. After three subsequent washings in PBS (3 minutes each) they were dehydrated in ethanol (reciprocal but otherwise as for rehydration). After air-drying of the slides and application of a hydrophobe frame, the hybridization solution containing the probes was added. The slides were placed in a moist chamber and were incubated over-night at 55 °C. Anti-DIG alkaline phosphatase FAB fragment was added to the slides after post hybridization washing and RNAse treatment of the slides. A solution of nitro blue tetrazolium (NBT) and 5-bromo-4-chloro-3-indolyl phosphate (BCIP) was used to visualize anti-DIG bound to DIG-labelled probes.

### Ethics statement

The studies were performed in compliance with requirements from the Norwegian Animal Research Committee, which were the requirements for cultivating parasites on fish. The cultivation was approved by the local research ethics committee at NIVA. The studies on the parasites presented in this paper were conducted on parasites detached from the fish. Studies in detached parasites do not require any specific permission.

The samples collected in the field were not collected from wild fish; they were all collected from farmed fish. The samples were collected with approval from the owner of the fish farm. The owner was informed that the samples would be used for research purposes. The samples were collected at a fish slaughtering plant from newly slaughtered fish. The studies did not involve endangered or protected species.

## Results

### Identification of *L*. *salmonis* AChE genes

Complete cDNA sequence encoding *L*. *salmonis ace1a* (GenBank KJ132368) and *ace1b* (GenBank KJ132369) were isolated from adult female *L*. *salmonis* samples. *Ace1a* has an open reading frame (ORF) of 1908 bp, which encodes a putative protein consisting of 635 amino acids. The 5’ untranslated region (UTR) is 729 bp whereas 3’UTR is 40 bp in length. *Ace1b* has an ORF of 1752 bp that codes for a protein of 583 amino acids. The 5’UTR and 3’UTR are 62 bp and 231 bp, respectively.

The deduced amino acid sequences from both *ace1a* and *ace1b* were aligned with 34 previously published AChEs from other insects, nematodes, arachnida and vertebrates using ClustalW alignment (listed in S1 Fig). Both the *L*. *salmonis* proteins exhibited high degree of similarity (83.5%) with each other. Moreover, both of these proteins showed highest similarity to AChE1 proteins from *Cimex lectularius* (69.7% with *ace1a* and 70% with *ace1b*) followed by *Bemisia tabaci* (69.6% with *ace1a* and 66.7% with *ace1b*), *Liposcelis entomophila* (68.2% with *ace1a* and 66.3% with *ace1b*), *Bombyx mori* (63.7% with *ace1a* and 63.4% with *ace1b*), *Bombyx mandarina* (64% with *ace1a* and 63.4% with *ace1b*), *Blattella germanica* (62.3% with *ace1a* and 64.3% with *ace1b*), *Chilo suppressalis* (62.3% with *ace1a* and 58.5% with *ace1b*), *Leptinotarsa decemlineata* (53.5% with *ace1a* and 54.2% with *ace1b*), and *Nephotettix cincticeps* (49.9% with *ace1a* and 50.6% with *ace1b*). However, only moderate level of cross similarity was observed between *L*. *salmonis* AChE1a and AChE1b with AChE2 from different species, ranging from about 53% (52.7% *ace1a* and 53.6% with *ace1b*) with *Blattella germanica* to about 50% (50.2% *ace1a* and 50.6% with *ace1b*) with *Chilo suppressalis*. Besides, both *L*. *salmonis* AChE1a and AChE1b showed only average percent of similary (50.2% *ace1a* and 50.9% with *ace1b*) to *Drosophila melanogaster* AChE and to *Torpedo californica* AChE (53.2% *ace1a* and 57.1% with *ace1b*), respectively. These observations strongly indicate that the *L*. *salmonis ace1a* and *ace1b* are two *ace1* paralogues.

**Fig 1:**
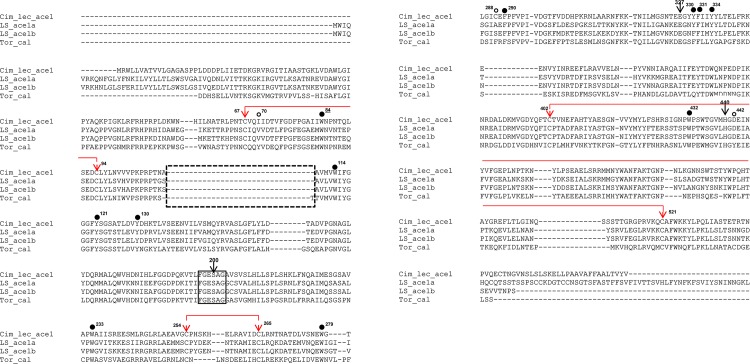
Alignment of Ls AChE1a and Ls AChE1b proteins with other AChE from other species. Alignment of AChE1a and AChE1b proteins from *Lepeophtheirus salmonis* (abbreviated to LS_ace1-A and LS_ace1-B) with AChE proteins from other species: *Cimex lectularius* AChE1; JN563927.1 (Cim__lec_ace1), and *Torpedo californica* AChE; CAA27169.1 (Tor_cal). By convention, numbering is that of *T*. *californica*. The three amino acids composing the catalytic triad (S200, E327 and H440) are indicated by arrows. The 14 conserved aromatic residues lining the active gorge are represented by circles. Out of these 14, 11 residues were present in both *L*. *salmonis* AChE1a and AChE1b (shown by filled circles), whereas the other 3 non conserved residues (shown by open circles) were absent in both the proteins of salmon louse. The choline binding site (W at 84) is underlined. Three interchain disulphide bridges are drawn between conserved C residues (shown by arrows). The solid box represents the canonical *FGESAG* motif, characteristic of the active site of cholinesterases. The dotted box represents the typical sequence insertion/deletion domain that easily distinguishes AChE1 and AChE2 proteins.

The amino acid alignment revealed that both *L*. *salmonis* AChE1a and AChE1b have the characteristic features of AChE, including the anionic choline binding site (W84 [*W115*]; the numbering of amino acids is based on the *Torpedo californica* AChE with the numbering in *L*. *salmonis* AChE1a given in brackets and italics), the three residues of the catalytic triad (S200 [*S230*], E327 [*E358*] and H440 [*H472*]), the six cysteines involved in three conserved disulphide bonds (C67-C94 [*C98-C125*], C254-C265 [*C284-C297*], C402-C521 [*C434-C556*]), the characteristic *FGESAG* motif surrounding the active serine and the 14 aromatic residues lining the active site gorge, 11 of which were well conserved and present in both *L*. *salmonis* AChE1a and AChE1b (Fig 1). This includes the acyl pocket residues W233 [*W263*], F290 [*F321*] and F331 [*F362*]) that accommodate the acyl moiety of the active site. In addition, the residues that form the oxyanion hole, helping to stabilize the tetrahedral molecule during catalysis (G118 [*G149*], G119 [*G150*] and A201 [*A231*]), were also present in both the proteins. The three non-conserved amino acids (70, 121 and 442) were substituted by other amino acids in both the salmon lice AChE proteins. The alignment for the *L*. *salmonis* AChE1a and AChE1b with *Cimex lectularius* AChE1 and the *T*. *californica* AChE is presented in Fig 1. The full alignment with 34 typical AChE proteins from other species (insects, nematods, arachnida and vertebrates) is presented in S1 Fig.

Interestingly, both *L*. *salmonis* AChE1a and AChE1b and most of the AChEs from other species, except *T*. *californica*, have aspartic acid at 442 [*474*] instead of tyrosine. Moreover, F290 [*F321*] is present and F288 is substituted by another amino acid in both *L*. *salmonis* AChE1a [*A319*] and AChE1b [*S319*], a characteristic property of all invertebrate AChEs, explaining a wider substrate specificity than vertebrate AChEs [35].

At the C terminal of the proteins, *L*. *salmonis* AChE1a has the putative hydrophobic peptide when compared to *Torpedo californica* and *Drosophila melanogaster* acetylcholinesterase sequences (S2 Fig). But no such hydrophobic peptide was found in *L*. *salmonis* AChE1b (S2 Fig). The site of cleavage of the hydrophobic peptide has already been determined in *Torpedo californica* [36] and *Drosophila melanogaster* [37]. Comparing amino acid sequences of *L*. *salmonis* AChE1a and *L*. *salmonis* AChE1b with acetylcholinesterases from *Torpedo californica* and *Drosophila melanogaster*, C595 was considered to be the most probable cleavage site of the hydrophobic peptide in *L*. *salmonis* AChE1a (S2 Fig), which corresponds to C615 in *Drosophila melanogaster* and C558 in *Torpedo californica*, respectively. However, no free cysteine residue (that could be the potential cleavage site of the hydrophobic peptide) is present in C terminal of *L*. *salmonis* AChE1b (S2 Fig).

Attempts to predict a potential glycosylphosphatidylinositol (GPI) modification site in *L*. *salmonis* AChE1a and *L*. *salmonis* AChE1b were inconclusive. Following the GPI prediction server (http://mendel.imp.ac.at/sat/gpi/gpi_server.html), neither *L*. *salmonis* AChE1a nor *L*. *salmonis* AChE1b had the GPI modification site. However, the best scoring amino acid in *L*. *salmonis* AChE1a was codon 600, close to the C-terminal. Interestingly, the similar inconclusiveness has been reported in AChE from *Haematobia irritans* [38], where the authors could find a potential GPI anchor site in the *Haematobia irritans* AChE using a DGPI program but the GPI prediction server showed no GPI modification site. Unfortunately, we couldn’t use the DGPI software on our proteins because the website is no longer available.

However, similar to *Haematobia irritans* AChE, the hydrophobic C-terminal peptide of *L*. *salmonis* AChE1a was identified as a transmembrane helix by the online modelling platform SOSUI (http://harrier.nagahama-i-bio.ac.jp/sosui/sosui_submit.html), between amino acids S597 and Y619 in *L*. *salmonis* AChE1a. This observation could suggest the possibility of *L*. *salmonis* AChE1a being the main synaptic enzyme in *L*. *salmonis*.

### Phylogenetic analysis of *L*. *salmonis* AChEs

**Fig 2:**
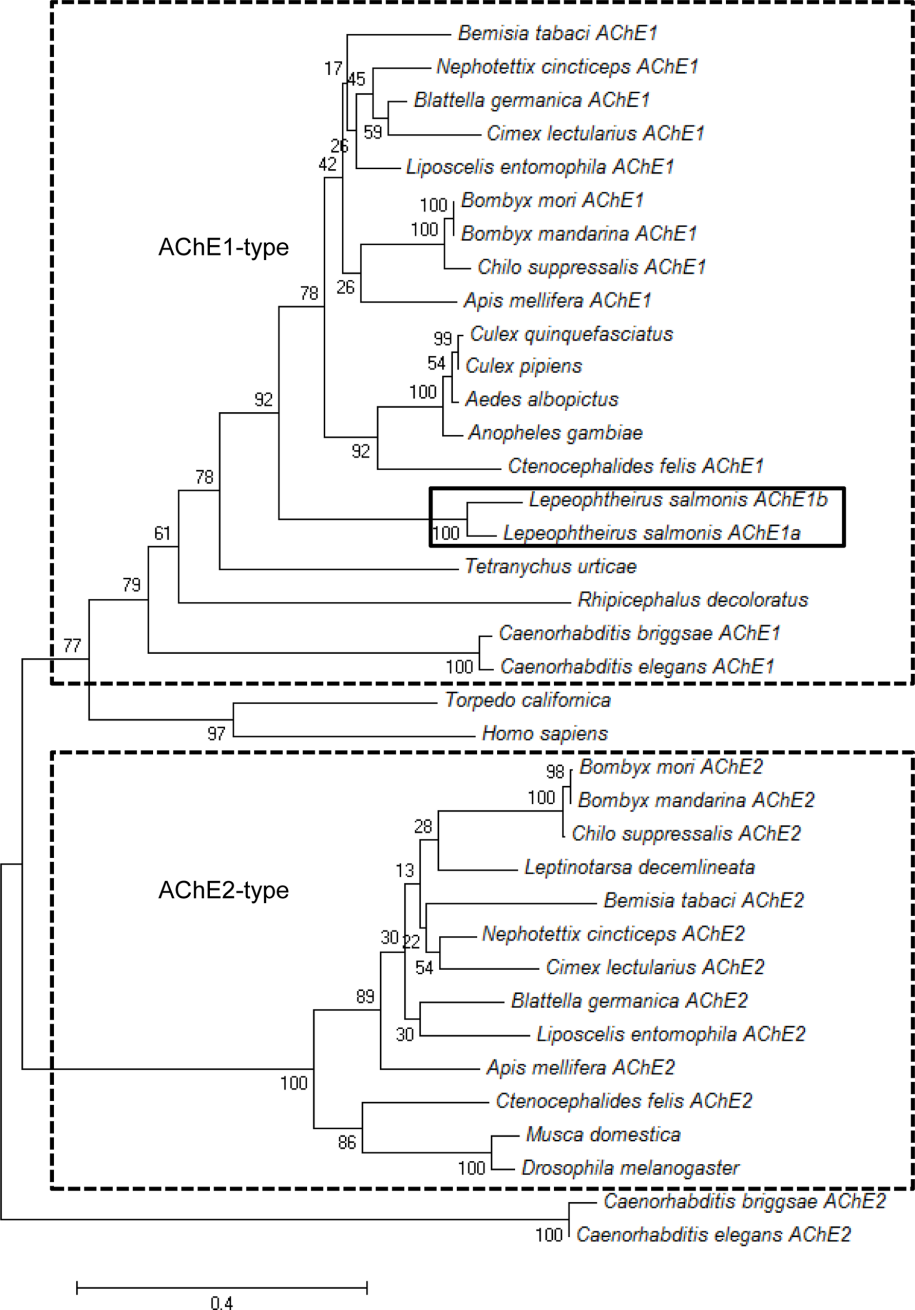
Phylogenetic tree. Phylogenetic relationship of *Lepeophtheirus salmonis* AChE1a and AChE1b with other acetylcholinesterases from Insecta, Nematoda, Arachnidae and Veterbrata is shown. The phylogenetic tree was constructed using a MUSCLE alignment at the Phylogeney.fr platform (http://www.phylogeny.fr/version2_cgi/index.cgi). The clustering of the AChE1 and AChE2-type of enzymes are indicated by boxes. The AChE1a and AChE1b of *L*. *salmonis* are also boxed. Branch support values are given in %. AChE1a and AChE1b were clustered with other AChE1 proteins and were clearly separated from AChE2 proteins in the phylogenetic tree.

A phylogenetic tree was constructed using the maximum likelihood method to the conserved regions of the *L*. *salmonis* proteins and other AChEs proteins from 34 different species deposited in GenBank. The phylogenetic tree showed that both salmon louse proteins clustered with other AChE1 proteins and they were clearly separated from AChE2 proteins that form a separate clad in the phylogenetic tree (Fig 2). In addition, *L*. *salmonis* AChE1a and AChE1b were grouped together in the phylogenetic tree, exhibited a high similarity (84% at the protein level) and were located very close to each other (genetic distance: 26.4 kbp). This clearly demonstrated that they are two close paralogues from a relatively recent duplication event.

### Genomic organization of *L*. *salmonis* AChEs

**Fig 3:**
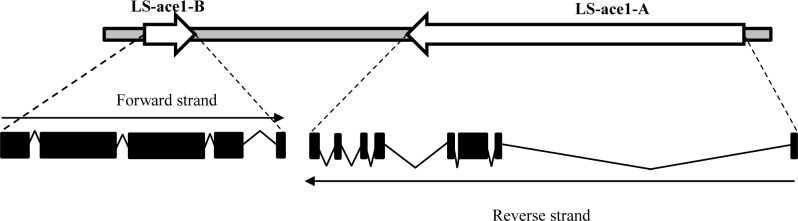
Genomic organization. *L*. *salmonis* assembly (sealouse.imr.no) was used to find the organization of the LS-ace1-A and *ace1b* LS-ace1-B in the *L*. *salmonis* genome. *Ace1a* is larger in size (48 kbp) with 8 exons compared to *ace1b* (3 kbp) with only 5 exons. The size of the exons (dark boxes) and intron (lines) are not at scale. The two genes were separated from each other by *a* genomic distance of 26.4 kbp and are encoded in opposite direction.

The genomic organization revealed that the two *L*. *salmonis* genes are present on the same super contig where they span around 76.9 kbp. The *L*. *salmonis* AChE1a and AChE1b are encoded on opposite strands and the distance between the two genes is about 26.4 kbp. The *L*. *salmonis ace1a* is large, consisting of 8 exons and span 48kbp, whereas *ace1b* consists of 5 exons and span 3079 bp. The main difference between the two genes is the large first intron in *ace1a* that is situated in the 5’-UTR of the gene (Fig 3).

### Three-dimensional modelling of *L*. *salmonis* AChEs

**Fig 4:**
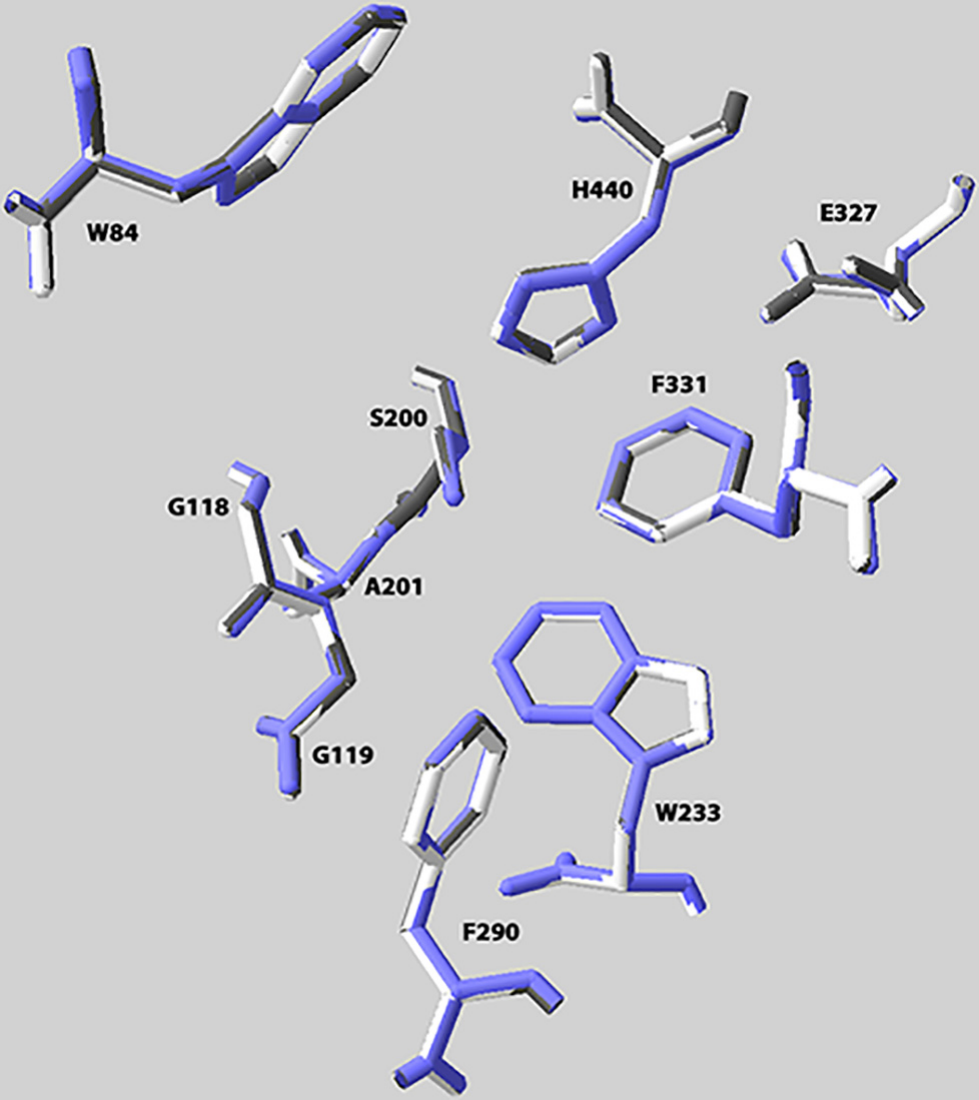
3D structure. Predicted three-dimensional positioning of ten functionally important essential amino acids in AChE1a (white) and AChE1b (grey) from *Lepeophtheirus salmonis* and the AChE template (PDB-ID 1qo9) from *Drosophila melanogaster* (blue) was used. SWISS MODEL in the automated mode [24] (http://swissmodel.expasy.org/) was used for modelling. The numbering is from *T*. *californica*. The catalytic triad amino acids S200, E327 and H440 were predicted to be in almost exactly the same positions in all proteins. The same goes for the important acyl pocket residues W233, F290, F331, the choline binding site W84 and the oxyanion hole residues G118, G119 and A201.

The 3D structures of the two *L*. *salmonis* proteins were predicted using native AChE from *Drosophila melanogaster* as template. The superimposed functionally important amino acids of the AChE template from *D*. *melanogaster* and the corresponding, modelled amino acids for *L*. *salmonis* AChE1a and AChE1b are presented in Fig 4. The catalytic triad amino acids S200, E327 and H440 were predicted to be in almost exactly the same positions in all proteins. The same goes for the important acyl pocket residues W233, F290, F331, the choline binding site W84 and the oxyanion hole residues G118, G119 and A201, indicating that both AChE1a and AChE1b were functionally active enzymes. The numbering of amino acids is by convention from the *Torpedo californica* amino acid sequence. The generated PDB-files have been included in the supporting information as S3 and S4 Files.

### Quantitative analysis of *L*. *salmonis ace1a* and *ace1b* in different life stages

**Fig 5:**
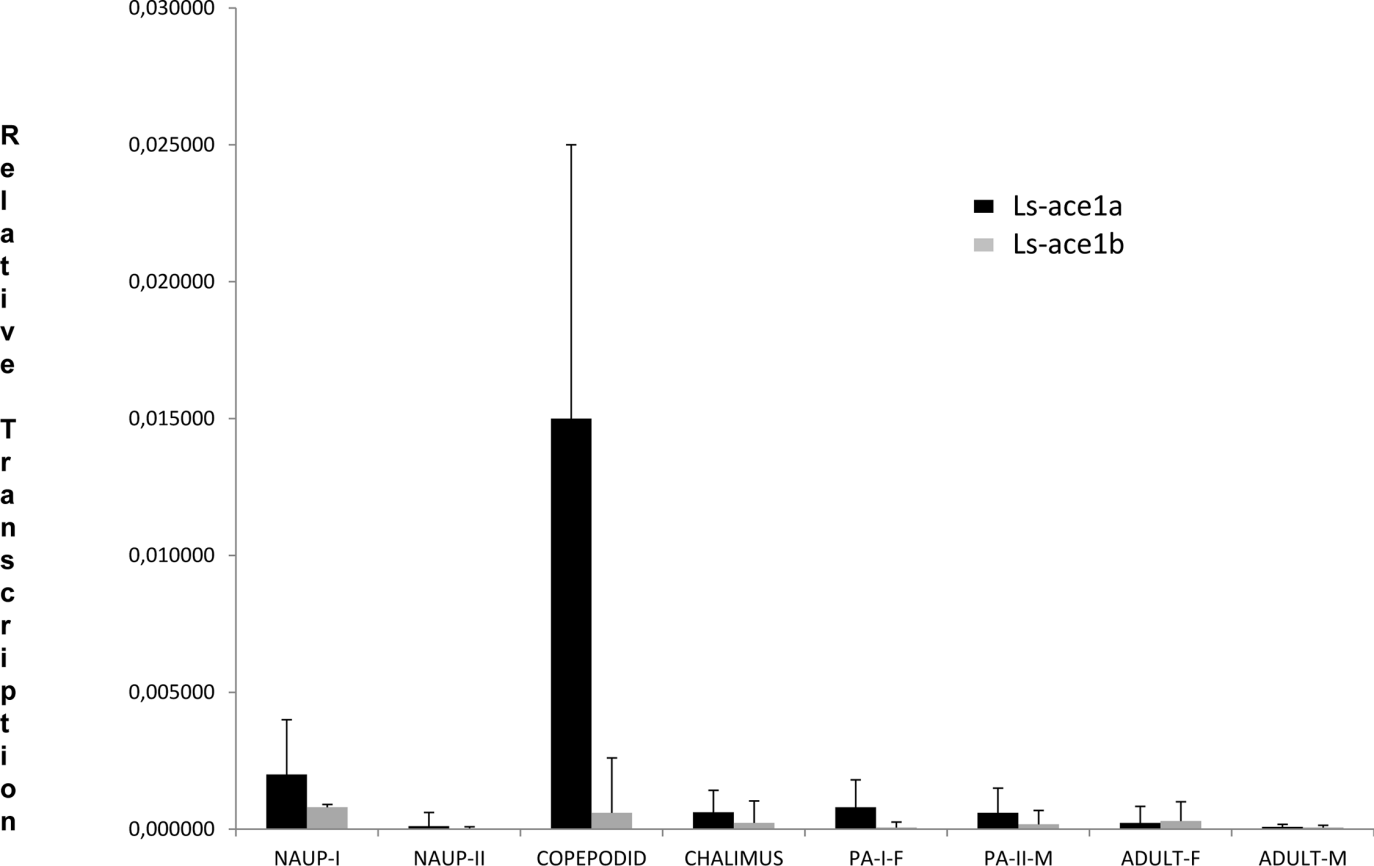
Fold difference in transcription between developmental stages. This figure displays the relative transcript levels of *ace1a* and *ace1b* at 7 different developmental stages of *Lepeophtheirus salmonis*. The relative expression level of *ace1a* was significantly higher than *ace1b*, especially at the copepodid (26 fold) stage and in preadult females (13 fold). The error bars indicate the SD (n = 3) and the experiment was performed in triplicates.

Quantitative real time PCR was performed to compare the expression pattern of *L*. *salmonis ace1a* and *ace1b* at 6 different (nauplius I, nauplius II, copepodid, chalimus, preadult and adult) developmental stages of the same batch. The relative expression level of *ace1a* was found to be significantly higher than *ace1b* (Fig 5), especially at the copepodid (26 fold) stage followed by preadult females (13 fold). The significantly higher transcription level of *ace1a* suggested that this is the predominantly expressed gene in *L*. *salmonis*.

The comparison of transcription levels of *ace1a* and *ace1b* in the cephalothorax and posterior body tissues of adult female *L*. *salmonis* revealed that expression of *ace1a* was higher (1.5 fold) than *ace1b* in the cephalothorax (data not shown). However, a lower level (-7 fold) of *ace1a* expression was observed in the posterior part compared to *ace1b*. The statistical significance could not be determined on this set due to the small sample size (n = 3).

### Molecular forms of *L*. *salmonis* AChEs in various tissues

The activity levels of *L*. *salmonis* AChE1a and AChE1b were evaluated and compared by Native PAGE gel electrophoresis (S3 Fig). One prominent band (band A) and one faint band (band C) was detected in the cephalothorax. On the other hand, three bands (band A, band B and band C) were detected in both the posterior body tissue and the homogenates from the whole body (S3 Fig). Predicted molecular weights of AChE1a and AChE1b were 71.72 kDa and 69.48 kDa, respectively, and their predicted isoelectric points (pI) were 6.77 and 5.1, respectively. Based on the predicted molecular weights and pI values, the most slowly migrating band (band A) appears to be AChE1a and the band (band B) observed only in the back body tissue could be AChE1b, whereas the band C could be another possible isoform of one of the enzymes, resulting from the posttranslational changes.

In addition, AChE was found to be active in both sensitive (Ls A) and resistant (parasites supplied from a site in middle Norway after a treatment failure) sea lice samples (S4 Fig). Preadult parasites were used for this experiment. Since the proportion of neural tissue compared to the body size is greater in early developmental stages, only one band (Band A), corresponding possibly to AChE1a, was observed (S4 Fig).

Taken together, the native PAGE gel electrophoresis and transcription levels of *ace1a* and *ace1b* at developmental stages suggested that *L*. *salmonis* AChE1a was predominantly expressed and possibly the more active enzyme in the cephalothorax tissues (head and ganglia), whereas AChE1b was mostly expressed and active in the posterior body tissue.

Western blotting using polyclonal antibodies, specific for *L*. *salmonis* AChE1a and AChE1b was conducted to confirm the results of Native polyacrylamide gel electrophoresis (S2 File). However, the level of non-specificity for both proteins was too high to conclude the results.

### In situ hybridization

**Fig 6:**
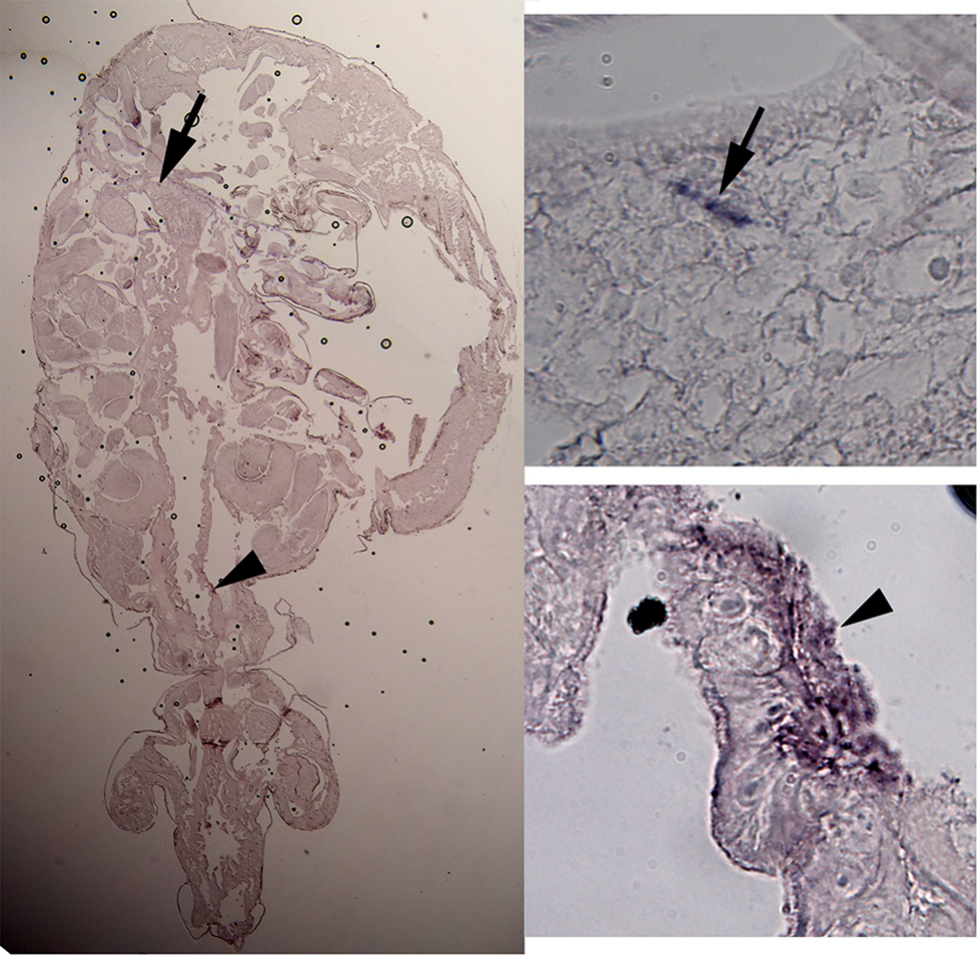
*In situ* hybridization. *In situ* hybridization in *ace1a* in the preadult salmon louse. Bound probe to mRNA fragments on a 3 μm coronal section appear as blue stain. The arrow points to staining in the central ganglion and the arrowhead indicates staining in the intestinal wall. Images on the right panels are a 10-fold magnification of the positive areas.

In the copepodid developmental stage there was staining for *ace1a* in ganglions and the intestinal wall. Due to the size of the specimens it was difficult to get representative neighbor sections which allows for comparison between the sense and anti-sense probes. In the preadult stage there was a significant staining for *ace1a* in the central ganglion and in the posterior part of the intestinal wall in the cephalothorax segment (Fig 6). In addition, staining in the intestinal wall was also apparent in the adult louse. The neuronal expression of the *ace1a* gene strengthened the hypothesis that AChE1a plays an important role in terminating ACh-generated signals at the postsynaptic level.

## Discussion

In the present study we identified the full length cDNA sequence of two genes coding for AChE in *L*. *salmonis* (*ace1a* and *ace1b*), followed by the detailed characterization of their structural properties.

The deduced amino acid sequences suggested that both genes possess typical properties of AChE, encoding for functional AChEs. Presence of two *ace* genes is an ancestral character that is common in most of the other arthropods, for example *A*. *gambiae* [39], *A*. *gossypii* [40], *C*. *pipiens* [30], *C*. *suppressalis* [41], *P*. *xylostella* [10], *H*. *assulta* [11], *B*. *germanica* [42], *A*. *mellifera* [28]. However, some arthropods are exception to this notion and only one *ace* (*ace2*) is present in *D*. *melanogaster* and *M*. *domestica* [39], which was attributed to the loss of one *ace* (*ace1*) during evolution.

The two *ace* genes in *L*. *salmonis* are highly similar to each other (83.5% in amino acid sequences). This is in contrast to most other arthropods where the two *ace* genes typically show moderate to low similarity, and the two AChEs are far more different from each other and fall in separate clads in the phylogenic tree. For example, 35.6%, 39% and 53% similarity has been reported between *C*. *lectularius*, *C*. *suppressalis* and *A*. *gambiae ace1* and *ace2*, respectively [9, 39, 41]. In *L*. *salmonis*, both the *ace* genes are in the same cluster as other *ace1* genes from other arthropods in the phylogenetic tree, and the two genes are closest relatives to each other (Fig 2), showing that they are two *ace1* paralogues. In addition, both the *L*. *salmonis* AChEs exhibited the typical feature of lacking 32 amino acids in the catalytic domain, which has been reported to be a common feature of all the insect *ace1* type genes [8].

The predicted 3D modelling structures of *L*. *salmonis* AChE1a and AChE1b supported the above observations quite well. Both AChE1a and AChE1b seemed to have highly similar 3D structures (comprising all the important features of the functional AChE), also indicating that both AChE1a and AChE1b were functionally active enzymes. The superimposed structure of the AChE template from *D*. *melanogaster* and the modelled structures for *L*. *salmonis* AChE1a and AChE1b are presented in Fig 4. The amino-acid similarity with the AChE protein from *D*. *melanogaster* was 44%, generally a suboptimal identity for 3D modelling. However, the RMS values for the fit between the modelled proteins and the template for the whole protein (RSM 0.25 for AChE1a and 0.27 for AChE1b) as well as for the ten functionally important amino acids (RSM 0.05 for both) were low, indicating that the models still were useful. This assumption was further supported by the superimposed positions of the ten highly conserved amino acids at the choline binding site, the catalytic triad, the acyl pocket and the oxyanion hole (Fig 4).

In other arthropods, significant differences have been found among the two AChEs, for example structural difference at the active gorge entrance and the conformation of the catalytic triad has been observed in the 3D structures of AChE1 and AChE2 of *B*. *germanica* [33]. This difference was considered to be responsible for the higher catalytic efficiency but lower substrate specificity for AChE2 compared to AChE1 in this species. Similar structural differences were observed in *A*. *mellifera* [28]. In addition, different configurations of the W84 residue forming the choline-binding site were observed in AChE1 and AChE2 proteins of *C*. *lectularius* [9].

The quite different and interesting observations in *L*. *salmonis* could be explained by two hypotheses. A: These two proteins are products of two different but very much homologous genes at different loci, which are the result of a recent duplication of an ancestral gene, or B: These two proteins could possibly be the products of two different alleles resulted from alternative splicing of the same gene. This puzzle was solved by the genomic organization of the two *ace* genes in *L*. *salmonis*. It supported the first hypothesis that the two ace genes are derived from a common ancestor and are located very close to each other in the *L*. *salmonis* genome with a genomic distance of 26.4 kbp between them, but are encoded in opposite direction (Fig 3), with *ace1a* being larger in size (48 kbp) compared to *ace1b* (3 kbp).

The transcription patterns of *L*. *salmonis ace1a* and *ace1b* suggested a higher expression of both *ace1a* and *ace1b* in early developmental stages (Fig 5) with a significantly higher expression of *ace1a* compared to *ace1b* (26 fold at copepodid, 8 fold at nauplius and 13 fold at preadult stage, respectively). The reason for the higher transcription level in the early developmental stages could be a higher density of neural tissues compared to the other developmental stages included in the present study. The higher expression of *ace1a* again suggests that *ace1a* could likely encode the predominant AChE in *L*. *salmonis*.

Similar observations have been made in other arthropods, for example in *B*. *germanica*, *ace1* had a 10 fold higher expression level compared to *ace2* [42]; in *C*. *lectularius*, transcription level of *ace1* was 1.7 fold to 5.5 fold higher than *ace2* in the tissues examined [43]. Based on this observation, it was concluded that *ace1* encodes the predominant AChE in these organisms. Exceptions have also been reported to this scenario, with *ace2* being the major synaptic enzyme in *A*. *mollifera* [28], in *D*. *melanogaste*r [14] and in *M*. *domestica* [16].

However, the specialization of one AChE (either AChE1or AChE2) as a neurological enzyme is a complex and not completely understood process. Even though the majority of reports suggest AChE1 with main catalytic properties, a recent study by Kim and Lee showed 33 insect species with only AChE2 as a predominantly expressed enzyme [19]. Interestingly, some species do not clearly fall in either of the category. For example, in *Calopteryx* spp. damselflies, both *ace1* and *ace2* are almost equally active, indicating the likely involvement of both the enzymes in the synaptic transmission [19].

Functionality of the two different AChEs in *L*. *salmonis* was demonstrated by the native PAGE gel electrophoresis (S3 Fig). The cephalothorax tissue was resolved in one darkly stained band (band A) and one faint band (band C), whereas the posterior tissue resolved in 3 bands (bands A, B and C), of which bands A and B were darkly stained compared to band C. Since the AChE1a was predicted to have higher pI value (pI = 6.77; less negatively charged) and larger molecular weight (71.72 kDa) than AChE1b (pI = 5.1, molecular weight = 69.48 kDa), the most slowly migrating band (band A) was most probably corresponded to AChE1a and the other darkly stained band (band B), observed only in posterior tissue, could be AChE1b, respectively. The faintly stained band (band C) could be a different molecular isoform resulting from post translational modification of AChE1a or AChE1b, which needs further elucidation. However, the presence of different molecular forms (e.g. hydrophilic form and amphiphilic form) is a common scenario and has been shown in other arthropods as well [1, 5, 8, and 42].

Western blotting would have further confirmed our native PAGE gel electrophoresis results. However, the high level of nonspecific binding of the antibodies produced against both *L*. *salmonis* AChE1a and AChE1b limited our attempt. Affinity purification of the antibodies was not useful in solving the problem.

The *in situ* hybridization demonstrated that *ace1a* was clearly expressed in the central ganglion and in intestinal tissues of preadult females (Fig 6). AChE is expected in the central nervous system of *L*. *salmonis*, and the *in situ* studies clearly indicated that AChE1a is a neurological form. The appearance in the intestinal wall was more surprising. AChE has though also been located in the honey bee gut by Western blotting [28]. AChE activity in the enteric nervous system has been described in mammals [44, 45].

In conclusion, two AChEs have been identified in *L*. *salmonis* and their molecular properties have been characterized. In contrast to most of the other arthropods, *L*. *salmonis* has two different forms of the *ace1* gene with no orthologous of the *ace2* gene. *Ace1a* was the predominantly expressed gene especially at the early life stages of *L*. *salmonis*, and was likely to be more active in the cephalothorax compared to *ace1b* in salmon lice. Since the head ganglion and most other ganglia are located in the cephalothorax and the proportion of neural tissue compared to the body size is greater in early developmental stages, *ace1a* could possibly be the gene encoding for the main functional AChE in *L*. *salmonis* with the major role in cholinergic synaptic transmission. Further insights into the physiological functions of *ace1b* are warranted to validate our findings. However, the elucidation of the cause of azamethiphos resistance in salmon lice being a mutation in the *ace1a* gene [46] serves as a substantial additional validation to the conclusions of the present study.

## Supporting Information

S1 Fig Alignment of Ls AChE1a and Ls AChE1b proteins with other typical AChE proteins from other species. (PDF)

S2 Fig Hydrophobic peptide. Alignment of Ls AChE1a and Ls AChE1b proteins with *Torpedo californica* AChE and *Drosophila melanogaster* AChE. The alignment corresponding to hydrophobic peptide sequences was manually edited. The site of cleavage (cysteine) of the hydrophobic peptide has been underlined in Ls AChE1a, *Torpedo californica* AChE and *Drosophila melanogaster* AChE. Ls AChE1b did not have the free cysteine residue that could serve as the site of cleavage of the hydrophobic peptide.
(TIFF)

S3 Fig Activity staining. Native polyacrylamide gel electrophoresis of *Lepeophtheirus salmonis* acetylcholinesterases from cephalothorax segment, posterior segment and whole body tissues of female adult lice. Protein samples (50 μg) were loaded on 8% polyacrylamide gel. After running, the gel was activity-stained to visualize AChE bands according to Lewis and Shuttle (29).
(TIF)

S4 Fig Activity staining in sensitive and resistant sea lice samples. Native PAGE was performed on sensitive and resistant sea lice samples. Both the sensitive (lane S) and resistant (lane R) samples showed active AChE. Since preadult parasites were used for the experiment, which have a higher proportion of neural tissue compared to the body size, only one band was observed. Based on S3 Fig (MW and pI of AChE1a and AChE1b), this band possibly corresponds to AChE1a.
(TIF)

S1 File Primers used for RACE PCR, TOPO TA Cloning and Quantitative analysis of *L*. *salmonis ace1a* and *ace1b*. (XLSX)

S2 File Western blot analysis. (DOCX)

S3 File PDB-file of the predicted 3D structure of the AChE1a in *Lepeophtheirus salmonis* using AChE from *Drosophila melanogaster* as template (PDB-ID: 1qo9). (PDB)

S4 File PDB-file of the predicted 3D structure of the AChE1b in *Lepeophtheirus salmonis* using AChE from *Drosophila melanogaster* as template (PDB-ID: 1qo9). (PDB)
