# Supplementary material for: Identification and Molecular Characterization of Two Acetylcholinesterases from the Salmon Louse, Lepeophtheirus salmonis
Source: PLoS One. 2015 May 4;10(5):e0125362. doi: 10.1371/journal.pone.0125362 (PMC4418574; doi:10.1371/journal.pone.0125362)
Supplement: S1 Fig — (PDF) [file pone.0125362.s001.pdf]

```
Bla_ger_ace1      ------------------------------------------------------------
Nep_cin_ace1      ------------------------------------------------------------
Lip_ent_ace1      ------------------------------------------------------------
Cim_lec_ace1      ------------------------------------------------------------
Bom_man_ace1      ------------------------------------------------------------
Bom_Mor_ace1      ------------------------------------------------------------
Chi_sup_ace1      ------------------------------------------------------------
Bem_tab_ace1      ------------------------------------------------------------
Api_mel_ace1      ------------------------------------------------------------
Cul_pip           ----------------------------------------------MEIRGLITRLLGPC
Cul_qui           ----------------------------------------------MEIRGLITRLLGPC
Aed_alb           ----------------------------------------------MEIRGLITRLLGPC
Ano_gam           ---------------------------------------MEIRGLLMGRLRLGRRMVPLG
Cte_fel_ace1      ------------------------------------------------------------
LS_ace1-A         ------------------------------------------------------------
LS_ace1-B         ------------------------------------------------------------
Tet_urt_ace1      ------------------------------------------------------------
Rhi_dec           ------------------------------------------------------------
Celeg_ace1        ------------------------------------------------------------
Cae_bri_ace1      ------------------------------------------------------------
Homosap           ------------------------------------------------------------
Tor_cal           ------------------------------------------------------------
Bom_man_ace2      ------------------------------------------------------------
Bom_Mor_ace2      ------------------------------------------------------------
Chi_sup_ace2      ------------------------------------------------------------
Lep_dec           ------------------------------------------------------------
Nep_cin_ace2      ------------------------------------------------------------
Cim_lec_ace2      ------------------------------------------------------------
Lip_ent_ace2      ------------------------------------------------------------
Bla_ger_ace2      --------------------------------------------------------MATQ
Api_mel_ace2      ------------------------------------------------------------
Bem_tab_ace2      ------------------------------------MATPATTRVVTQRQRREPCPRGTK
Dros              ------------------------------------------MAISCRQSRVLPMSLPLP
Mus_dom           MARSVRTPISPSSSSSSSRSSWSSPSSSFYSLLSSFKASLTRPSSSSSVAHHLAARNNDI
Cte_fel_ace2      ----------------------------------------------------------MY
Celeg_ace2        ------------------------------------------------------------
Cae_bri_ace2      ------------------------------------------------------------
```

```
Bla_ger_ace1      -------MDVGDLVGGLSVGSGDTDPLRDIHIREHVIKDHVHSHHGSG------------
Nep_cin_ace1      ------------------------------------------------------------
Lip_ent_ace1      ------------------------------------------------------------
Cim_lec_ace1      ------------------------------------------------------------
Bom_man_ace1      --MRVVLAALTALAARTLAGPHEHRARHHAPAP----------PQPYH------------
Bom_Mor_ace1      --MRVVLAALTALAARTLAGPHEHRARHHAPAP----------PQPYH------------
Chi_sup_ace1      --MRVVLAALTALAARALAGPHEHRARHHAPDHPLHFPAPANPAQPYR------------
Bem_tab_ace1      ------------------------------------------------------------
Api_mel_ace1      ------------------------------------------------------------
Cul_pip           HLRHLILCSLGLYSILVKSVHCRHHDIGSSVAHQLG--SKYSQ-----------------
Cul_qui           HLRHLILCSLGLYSILVQSVHCRHHDIGSSVAHQLG--SKYSQ-----------------
Aed_alb           HIRHLILCSLGIYSILVQSVHCRHHDIGSSTAHQLG--SKYAQ-----------------
Ano_gam           LLGVTALLLILPPSALVQGRHHELNNGAAIGSHQLS--AAAGVGLSSQSAQSGSLASGVM
Cte_fel_ace1      ---MQCGALLSLLAVVAQASRRREAPAAPAPAYADFDEQMG-------------------
LS_ace1-A         ------------------------------------------------------------
LS_ace1-B         ------------------------------------------------------------
Tet_urt_ace1      MVPMFNHNINHFNNVIVTTLTHHQYTNSRCNGNNNVIKSITN------------------
Rhi_dec           ------------------------------------------------------------
Celeg_ace1        ------------------------------------------------------------
Cae_bri_ace1      ------------------------------------------------------------
Homosap           ------------------------------------------------------------
Tor_cal           ------------------------------------------------------------
Bom_man_ace2      -MINYGKIVFTKLLLCVLISGTFA------------------------------------
Bom_Mor_ace2      -MINYGKIVFTKLLLCVLMSGTFA------------------------------------
Chi_sup_ace2      -MSRNIKIVFTKLLLCFFVSGAFG------------------------------------
Lep_dec           -----MGQLSILCLFVTVCASVCG------------------------------------
Nep_cin_ace2      -MARLRFSTLSLSLLVAVATQPQP------------------------------------
Cim_lec_ace2      -MSPWIGCVLAVVIGLAQGRPYSG------------------------------------
Lip_ent_ace2      ----MSRYSCTVIVISILVGHVWG------------------------------------
Bla_ger_ace2      CLLILAGCICTAMAIGAGSKPHFN------------------------------------
Api_mel_ace2      --MTTRILLLFFLLLSSCTRPSRGNA----------------------------------
Bem_tab_ace2      ELWPLALTLCAVLGLAAGHPSHRKHHG---------------------------------
Dros              LTIPLPLVLVLSLHLSGVCG----------------------------------------
Mus_dom           CRGLFATLVILLRMSALTSA----------------------------------------
Cte_fel_ace2      HLTSILVFSLLSISTRVDGS----------------------------------------
Celeg_ace2        ------------------------------------------------------------
Cae_bri_ace2      ------------------------------------------------------------
```

```
Bla_ger_ace1      --------------------GGDPSYMSRGDPSIVGRGEPMDMGRRDPPVDFGRRHSIDM
Nep_cin_ace1      --------------------------MVWLCPVYVLVCTMTCTCGFAATLRHARHQAAPP
Lip_ent_ace1      ----------------------------------MEYGERSSDILADGSLDLSRKDRVEF
Cim_lec_ace1      ------------------------------------------------------------
Bom_man_ace1      --------------------GHGEAVRYNPELDTILPRLEDHETSSKRASDAETSSKRTK
Bom_Mor_ace1      --------------------GHGEAVRYNPELDTILPRLEDHETSSKRASDAETSSKRTK
Chi_sup_ace1      --------------------GHGEAARYNPELDTILPRVEDHETSSKRAKLDDAETSSKR
Bem_tab_ace1      ------------------------MDFDHLPLRASPETDQLRNPRHGFGFRDGISDEGLN
Api_mel_ace1      -----------------------MPRNRNSLTKFEVLMVLSLTVLVQLDVCRCTPSHRSR
Cul_pip           ----------SSSLSSSSQSSSSLAEEATLNKDSDAFFTPYIGHGDSVRIVDAELGTLER
Cul_qui           ----------SSSLSSSSQSSSSLAEEATLNKDSDAFFTPYIGHGDSVRIVDAELGTLER
Aed_alb           ----------SSSLSSSSQSSSSLVEEPALNKDSDAFFTPYIGHGDSVRIVDAELGTLER
Ano_gam           SSVPAAGASSSSSSSLLSSSAEDDVARITLSKDADAFFTPYIGHGESVRIIDAELGTLEH
Cte_fel_ace1      ----------------------------PELPDEHGIYSPYMGHGESVRVLDPDLGTLEL
LS_ace1-A         --------------------------------------------------------MWIQ
LS_ace2-B         --------------------------------------------------------MWIQ
Tet_urt_ace1      --------------------SILKSVTVFTVKTLWNHLLVPIVVILLFQSSANVFSSALP
Rhi_dec           ----------------------------------------MLHENLASCHLTLLALLVCG
Celeg_ace1        ------------------------------------------------------------
Cae_bri_ace1      ------------------------------------------------------------
Homosap           -----------------------------------------------------MRPPQCL
Tor_cal           ------------------------------------------------------------
Bom_man_ace2      -------------------------------RSWANHHDTTTSTTQTTPTT---------
Bom_Mor_ace2      -------------------------------RSWANHHDTTTSTTQTTPTT---------
Chi_sup_ace2      -------------------------------RSWANHHDTTTSTTQTTPTT---------
Lep_dec           -------------------------------YSWP---------SDETTTK---------
Nep_cin_ace2      -------------------------------STPRTLHSNDHNHGFLNEHKHSHAHAYKS
Cim_lec_ace2      -------------------------------PTSERTHTPPR------------------
Lip_ent_ace2      -------------------------------APSWSRESLSLIN----------------
Bla_ger_ace2      -------------------------------SSSASSTPHSTIGGGSSPNT---------
Api_mel_ace2      ------------------------VPSSQRG-----------------------------
Bem_tab_ace2      -------------QTSHLNNHKNHNFDEDHYASFAAYQEPSNKTGTSNGDRKFKAGEEER
Dros              ------------------------------------------------------------
Mus_dom           ------------------------------------------------------------
Cte_fel_ace2      ------------------------------------------------------------
Celeg_ace2        -----------------------------------------------------MRAPVIG
Cae_bri_ace2      -----------------------------------------------------MRAPVNR
```

```
Bla_ger_ace1      DRVGHGGDPLDLAHESRMSEEGSGHGMQDDPLVIDTKKGKVRGITLTAATGKLVDAWLGI
Nep_cin_ace1      PDRLSPLDIPSRYEMDMTDDVVEDAPAPEDPLLIHTLKGKVRGQTMTAATGKLVDAWLGI
Lip_ent_ace1      GSRDKDIE---REREAKDDYHHEETKAEEDPLEIMTMKGKVRGTTLVAGNGKLVDAWLGI
Cim_lec_ace1      --------MRWLLVATVVLGAGASPPLDDDPLIIETDKGRVRGITIAASTGKLVDAWLGI
Bom_man_ace1      YEERFYSNHERAAELMADEPVSEKGDEED-PLVIRTRKGKVRGITLTSATGKKVDAWFGI
Bom_Mor_ace1      YEERFYSNHERAAELMADEPVSEKGDEED-PLVIRTRKGKVRGITLTSATGKKVDAWFGI
Chi_sup_ace1      DDDRFYSNHERIDDEGFLADEPQPGPEDDDPLIVRTRKGRVRGITLTAATGKKVDAWFGI
Bem_tab_ace1      FRHSEHEGERSKYKGAEAEEEMMADEGDNDPLVVQTTKGKVRGTTLTAATGKQVDAWLGI
Api_mel_ace1      HHADMSYEDTMSFRSGEELPRPAALNSNDDPLIVQTRKGKVRGKTMTATTGKEVDAWFGI
Cul_pip           EHIHSTTTRRRGLTRR----ESSSDATDSDPLVITTDKGKIRGTTLEAPSGKKVDAWMGI
Cul_qui           EHIHSTTTRRRGLTRR----ESSSDATDSDPLVITTDKGKIRGTTLEAPSGKKVDAWMGI
Aed_alb           EHVHSTTTRRRGLTRR----ESSSDANDNDPLLITTDKGKVRGLTLEAPSGKKVDAWLGI
Ano_gam           VHSG-ATPRRRGLTRR----ESNSDANDNDPLVVNTDKGRIRGITVDAPSGKKVDVWLGI
Cte_fel_ace1      GEEREAKRHREYRSSTGKTRRESSRHPEDDPLVVRTNKGLVRGTTLTSSTGKHVDAWLGI
LS_ace1-A         VRKQNFGLYFNKILVYLLTLSWSVGAIVQDNLVITTKKGKIRGVTLKSATNREVDAWYGI
LS_ace1-B         VRKHNLGLSFERILVYLLTLSWSLGSIVQEDLVITTRKGKIRGVTLKSATNKEVDAWYGI
Tet_urt_ace1      HSEINSLYADGPSFSSSFNSEHHHHHHHNDPLVVLTKKGYVRGRSVVSPTGKPVDAFLGI
Rhi_dec           GVVLRCLSIEPEEDAPNRVEDQDADEDHVETVVVQTAKGLVKGFIARSPLGKTVRVLYGI
Celeg_ace1        ------------MRNSLLFFIFLPSTILAVDLIHLHDGSPLFGEEVLSQTGKPLTRFQGI
Cae_bri_ace1      ------------MRYSLLFFIFLPCVITAVDLIHLHDGSPLFGEEVLSQTGKPLTRFLGI
Homosap           LHTPSLASPLLLLLLWLLGGGVGAEGREDAELLVTVRGGRLRGIRLKTPGG-PVSAFLGI
Tor_cal           --------------------------DDHSELLVNTKSGKVMGTRVPVLSS-HISAFLGI
Bom_man_ace2      --------------------SPVPKNIHNDPLIVETKSGLIKGY-AKTVMGREVHIFTGI
Bom_Mor_ace2      --------------------SPVPKNIHNDPLIVETKSGLIKGY-AKTVMGREVHIFTGI
Chi_sup_ace2      --------------------SPLPKNIHSDPLIVETKSGLIKGY-AKTVMGREVHIFTGI
Lep_dec           --------------------PSQFKDFHTDPLVVETTSGLVRGY-SKTVLGREVHVFTGI
Nep_cin_ace2      HDRAHNTHAQFAEATGPASTPSGGTPKHGDPLIVETTSGLVRGL-SKTVLGREVHVFTGI
Cim_lec_ace2      -------------------------AEDLDPMIVKTRSGLVRGM-AKSVLDREVHVFTGI
Lip_ent_ace2      --------------------TTASRDYHMDPLVVETTTGLVKGV-SKMVLDREVHVFYGI
Bla_ger_ace2      --------------------NPGKLDYHDDPLVVETQSGLVRGS-ARVVLGKEVHIFTGI
Api_mel_ace2      -------------------------NVHNDPLVVETTSGLVRGF-PRTVLDKEVHVFYGI
Bem_tab_ace2      KYIFDGQRRFKSLEHERHTHADYEESFVNDPLVVRTKSGLIRGV-EKQVMGHKVHVFTGI
Dros              ---------------------------VIDRLVVQTSSGPVRGR-SVTVQGREVHVYTGI
Mus_dom           ---------------------------MTDHLTVQTTSGPVRGR-SVTVQGRDVHVFTGI
Cte_fel_ace2      ---------------------------HQDPLTVTTTSGLIRGR-ARIVLGREVHVFTGI
Celeg_ace2        RHLTYHVFCQFALVTLFIVRRIEPRSIVRGDHVVHTPLGTIRGV-GQTFDGAKVSAFLGV
Cae_bri_ace2      RHLIYHVFYRFLLLAALFTKYVETRSIVRGDHVVHTPLGTIRGV-GQTFDGAKVSAFLGV
```

```
                                                    67v  o70           ●84
Bla_ger_ace1      PYAQKPLGPLRFRHPRPVDRWDKHGEIYNATKMPNSCVQIIDTVFGDFPGALIWNPNTPL
Nep_cin_ace1      PYAQKPLGPLRFKHPRPPDRWD---YVYNATKQPNSCVQIFDTVFGDFSGAMMWNPNTQI
Lip_ent_ace1      PYAQKPLGNLRFRHPRPVERWEG---VLNTTKLPNSCMQILDTVFGDFPGATMWNPNTPL
Cim_lec_ace1      PYAQKPIGKLRFRHPRPLDKWN---HILNATRLPNTCVQIIDTVFGDFPGAIIWNPNTQL
Bom_man_ace1      PYAQKPMGDLRFRHPRPVEDWG--DEILNTTTLPHSCVQIVDTVFGDFPGAMMWNPNTDM
Bom_Mor_ace1      PYAQKPMGDLRFRHPRPVEDWG--DEILNTTTLPHSCVQIVDTVFGDFPGAMMWNPNTDM
Chi_sup_ace1      PYAQKPIGDLRFRHPRPAESWG--EEILNTTTLPHPCVQIIDTVFGDFPGAMMWNPNTDM
Bem_tab_ace1      PYAQKPIGALRFRHPRPIDKWEG---ILNATKMPNSCTQIVDTVFGDFAGSAMWNPNTPM
Api_mel_ace1      PYAQKPLESLRFRHPRPAERWSG---ILNATTLPNSCVQILDTVFGEFAGATMWNPNTPL
Cul_pip           PYAQPPLGPLRFRHPRPAERWT---GVLNATKPPNSCVQIVDTVFGDFPGATMWNPNTPL
Cul_qui           PYAQPPLGPLRFRHPRPAERWT---DRG--------------HVFGDFPGATMWNRNTPL
Aed_alb           PYAQPPLGPLRFRHPRPVEKWT---GVLNATTPPNSCVQIVDTVFGDFPGATMWNPNTPL
Ano_gam           PYAQPPVGPLRFRHPRPAEKWT---GVLNTTTPPNSCVQIVDTVFGDFPGATMWNPNTPL
Cte_fel_ace1      PYGRNPSGALRFRHPRPADPWQ---GVLNATSPPNTCVQIVDTLFGDFPGATMWNPNTPI
LS_ace1-A         PYAQPPVGNLRFRHPRHIDAWEG---IKETTRPPNSCIQVVDTLFPGFEGAEMWNVNTEP
LS_ace1-B         PYAQPPVGNLRFRHPKDINAWDG---MKETTKHPNSCIQVVDTFFPGFEGSEMWNTNTEQ
Tet_urt_ace1      RYAKPPTGKFRFRRPKPIDSWQG---IFNATSFPGACYQVNDTFFGNFMGATEWNPNVPL
Rhi_dec           PYAKPPTGKRRFDRAESIEEPWT--DVFDATVKPNSCFQVLDTLYGNFSGSTMWNANTEM
Celeg_ace1        PFAEPPVGNLRFKKPKPKQPWRIP---LNATTPPNSCIQSEDTYFGDFYGSTMWNANTKL
Cae_bri_ace1      PFAEPPIGNLRFRKPKPKQPWRIP---FNATTPPNSCIQSEDTYFGDFYGSTMWNPNTKL
Homosap           PFAEPPMGPRRFLPPEPKQPWSG---VVDATTFQSVCYQYVDTLYPGFEGTEMWNPNREL
Tor_cal           PFAEPPVGNMRFRRPEPKKPWSG---VWNASTYPNNCQQYVDEQFPGFSGSEMWNPNREM
Bom_man_ace2      PFAKPPLGPLRFRKPVPIEPWHG---VLEANLMPNSCYQERYEYFPGFEGEEMWNPNTNI
Bom_Mor_ace2      PFAKPPLGPLRFRKPVPIEPWHG---VLEANLMPNSCYQERYEYFPGFEGEEMWNPNTNI
Chi_sup_ace2      PFAKPPLGPLRFRKPVPIDPWHG---VLEATAMPNSCYQERYEYFPGFEGEEMWNPNTNI
Lep_dec           PFAKPPIEQLRFKKPVPIDPWHG---ILDATKQPNSCFQERYEYFPGFEGEEMWNPNTNI
Nep_cin_ace2      PFAKPPVGPLRFRRPVPVDPWHG---VYDATTLSNSCYQERYEYFPGFEGEEMWNPNTNI
Cim_lec_ace2      PFAKPPVGPLRFRKPVPVENWHG---ILDATTLPNSCHQQRYEYFPGFEGEEMWNPNTNM
Lip_ent_ace2      PFAKPPAGPLRFRRPVPIDPWHG---VFDATTLPNSCYQERYEYFPGFEGEEMWNPNTNI
Bla_ger_ace2      PFAKPPVGPLRFRRPVPVEPWHG---VLDATALPNSCYQERYEYFKGFEGEEMWNPNTNV
Api_mel_ace2      PFAKPPIGPLRFRKPLPIEPWHG---VLNATVLPNSCYQERYEYFPGFPGEEMWNPNTNI
Bem_tab_ace2      PFAKPPVGMLRFRKPVEIDPWRG---VLNATSLPNSCYQERLEYFPGFQGEEMWNPNTNI
Dros              PYAKPPVEDLRFRKPVPAEPWHG---VLDATGLSATCVQERYEYFPGFSGEEIWNPNTNV
Mus_dom           PYAKPPVDDLRFRKPVPAEPWHG---VLDATRLPATCVQERYEYFPGFSGEEIWNPNTNV
Cte_fel_ace2      PYAKPPVGPLRFRKPVPAEPWHG---VLDATHPPNCCVQERYEYFQGFQGEELWNPNTNI
Celeg_ace2        PYAKPPIGSRRFKMAEMIDRWSG---ELEARTLAKTCYLTIDSAFPQFPGAEMWNPPGAI
Cae_bri_ace2      PYAKPPVGSRRFKMAEMIDRWSG---ELEARTLAKTCYLTIDSAFPQFPGAEMWNPPGAI
```

```
      94                                                     114
Bla_ger_ace1    SEDCLYINVVVPKPRPKNA---------------------------------AVMVWIFG
Nep_cin_ace1    SEDCLYLNVVAPKPRPSNA---------------------------------AVMVWVFG
Lip_ent_ace1    SEDCLYINVVAPKPRPKKA---------------------------------AVMVWIFG
Cim_lec_ace1    SEDCLYLNVVVPKPRPTNA---------------------------------AVMVWIFG
Bom_man_ace1    QEDCLYINIVTPRPRPKNA---------------------------------AVMLWVFG
Bom_Mor_ace1    QEDCLYINIVTPRPRPKNA---------------------------------AVMLWVFG
Chi_sup_ace1    QEDCLYINIVSPRPRPKNA---------------------------------AVMLWVFG
Bem_tab_ace1    SEDCLYINVITPKPRPRNA---------------------------------AVMVWIFG
Api_mel_ace1    SEDCLYVNVVVPRPRPTNA---------------------------------AVMVWIFG
Cul_pip         SEDCLYINVVVPRPRPKNA---------------------------------AVMLWIFG
Cul_qui         SEDCLYINVFVPRPRPKNA---------------------------------AVMLWIFG
Aed_alb         SEDCLYINVVVPHPRPKNS---------------------------------AVMLWIFG
Ano_gam         SEDCLYINVVAPRPRPKNA---------------------------------AVMLWIFG
Cte_fel_ace1    SEDCLYVNVVAPKPRPTNG---------------------------------PVMLWIFG
LS_ace1-A       SEDCLYLSVHVPKPRPTGS---------------------------------AVLVWIYG
LS_ace1-B       SEDCLYLSVHAPKPRPTKS---------------------------------AVLVWIYG
Tet_urt_ace1    DEDCLSVNIWVPRPRPKSA---------------------------------AVLLWIYG
Rhi_dec         SEDCLKLNVWTPGPSASSGGRP-----------------------------LAVLVWIYG
Celeg_ace1      SEDCLYLNVYVPGKVDPNKK-------------------------------LAVMVWVYG
Cae_bri_ace1    SEDCLYLNVYVPGKVDPNKK-------------------------------LAVMIWVYG
Homosap         SEDCLYLNVWTPYPRPTSP--------------------------------TPVLVWIYG
Tor_cal         SEDCLYLNIWVPSPRPKS---------------------------------TTVMVWIYG
Bom_man_ace2    SEDCLYLNIWVPQHLRVRHHQ---------------------DKPLAERPKVPILVWIYG
Bom_Mor_ace2    SEDCLYLNIWVPQHLRVRHHQ---------------------DKPLAERPKVPILVWIYG
Chi_sup_ace2    SEDCLYLNIWVPQHLRVRHHQ---------------------KKPLTEKPKVPILVWIYG
Lep_dec         SEDCLYLNIWVPQRLRIRHHA---------------------DKPTIDRPKVPVLIWIYG
Nep_cin_ace2    SEDCLYLNIWVPQRLRIRHKSS--------------------SEENTYRQKVPVLIWIYG
Cim_lec_ace2    SEDCLYLNIWVPQKIRLRHHN---------------------AQDRYNKPKVPVLIWIYG
Lip_ent_ace2    SEDCLYLNIWVPQRVRLRHHGGS-------------------QQDERHPHKVPMLIWIYG
Bla_ger_ace2    SEDCLYLNIWVPQRVRLRHSG--------------------------ERPKVPILVWIYG
Api_mel_ace2    SEDCLYLNIWVPQKYRLRHKGDGSPG----------------GNGGPRNGLLPLLVWIYG
Bem_tab_ace2    SEDCLYLNLWVPQKMRLRHRR-------------------------HIHQKAPVLIWIYG
Dros            SEDCLYINVWAPAKARLRHGRGANGGEHPNGKQADTDHLIHNGNPQNTTNGLPILIWIYG
Mus_dom         SEDCLFMNIWAPAKARLRHGRGTNGGEHSS--KTDQDHLIHSATPQNTTNGLPILIWIYG
Cte_fel_ace2    SEDCLYLNVFAPARLKGGGQEGQ---------------------------GLPMLIWIYG
Celeg_ace2      SEDCLNMNIWVPEDHDGS-----------------------------------VMVWIYG
Cae_bri_ace2    SEDCLNMNIWVPEDHDGS-----------------------------------VMVWIYG
```

```
                  ●121      ●130
Bla_ger_ace1      GGFYSGSATLDVYDHKTLVSEENVIIVSMQYRVASLGFLFFD--------TIDVPGNAGL
Nep_cin_ace1      GGFYSGSATLDVYDPRILVSEENVIYVSMQYRVASLGFLFFD--------TPEVPGNAGL
Lip_ent_ace1      GGFYSGTATLDVYDPKTLVSEEKVIVVSMQYRIASLGFLFFD--------TPDVPGNAGL
Cim_lec_ace1      GGFYSGSATLDVYDHKTLVSEENVILVSMQYRVASLGFLYLD--------TADVPGNAGL
Bom_man_ace1      GGFYSGTATLDVYDPKILVSEEKVVYVSMQYRVASLGFLFFD--------TADVPGNAGL
Bom_Mor_ace1      GGFYSGTATLDVYDPKILVSEEKVVYVSMQYRVASLGFLFFD--------TADVPGNAGL
Chi_sup_ace1      GGFYSGTATLDVYDPKIMVSEEKIVYVSMQYRVASLGFLFFD--------TPDVPGNAGM
Bem_tab_ace1      GGFYTGTATLDIYDYKILASEENVILVSMQYRITCLGFLYFD--------TQDVPGNAGL
Api_mel_ace1      GGFYSGSATLDVYDHKTLVSEEKVILVSMQYRVASLGFLYFG--------TPDVPGNAGL
Cul_pip           GGFYSGTATLDVYDHRTLASEENVIVVSLQYRVASLGFLFLG--------TPEAPGNAGL
Cul_qui           GGFYSGTATLDVYDHRTLASEENVIVVSLQYRVASLGFLFLG--------TPEAPGNAGL
Aed_alb           GGFYSGTATLDVYDHRTLASEENVIVVSLQYRVASLGFLFLG--------TPEAPGNAGL
Ano_gam           GGFYSGTATLDVYDHRALASEENVIVVSLQYRVASLGFLFLG--------TPEAPGNAGL
Cte_fel_ace1      GGFYSGSSTLDVYDPKTLAAEEGVLVVSMQYRVASLGFLFLG--------TPDAPGNAGL
LS_ace1-A         GGFYSGTSTLEVYDPRVLVSEENIIFVAMQYRVASLGFLFFD--------TEDVPGNAGL
LS_ace1-B         GGFYSGTSTLELYDPRVLVSEENIIFVGIQYRVASLGFLFFD--------TEDVPGNAGL
Tet_urt_ace1      GGFWSGSSSLDFYDGSVLAGEESIIFVSINYRVASLGFIFFD--------TSDAPGNAGL
Rhi_dec           GGFYSGTSTLDVYDARTLVSEENVVVVAMNYRVASLGFLSFG--------NETLPGNAGL
Celeg_ace1        GGFWSGTATLDVYDGRILTVEENVILVAMNYRVSIFGFLYMN--------RPEAPGNMGM
Cae_bri_ace1      GGFWSGTSTLDVYDGRILTVEENVILVAMNYRVSIFGFLYMN--------RSEAPGNMGM
Homosap           GGFYSGASSLDVYDGRFLVQAERTVLVSMNYRVGAFGFLALP-------GSREAPGNVGL
Tor_cal           GGFYSGSSTLDVYNGKYLAYTEEVVLVSLSYRVGAFGFLALH-------GSQEAPGNVGL
Bom_man_ace2      GGYMSGTATLDLYKADIMASTSDVIVASMQYRVGAFGFLYLNKYFS-PG-SEEAPGNMGL
Bom_Mor_ace2      GGYMSGTATLDLYKADIMASTSDVIVASMQYRVGAFGFLYLNKYFS-PG-SEEAPGNMGL
Chi_sup_ace2      GGYMSGTATLDLYKADIMASSSDVIVASMQYRVGAFGFLYLNKYFS-AG-SEEAPGNMGL
Lep_dec           GGYMSGTATLDVYDADIIAATSDVIVASMQYRLGSFGFLYLNRYFP-RG-SDETPGNMGL
Nep_cin_ace2      GGYMSGTATLDIYDADMVAATSDVIVASMQYRVGAFGFLYLSPELP-PG-SEEAPGNLGL
Cim_lec_ace2      GGYMSGTATLDVYDGLMVAATSDVIVASMQYRIGAFGFLYLEPLVK-TG-SNDAPGNMGL
Lip_ent_ace2      GGFMSGTSTLDVYDADIVAATSDVIVASMQYRIGAFGFLYLAPYSKNKD-NDEAAGNMGL
Bla_ger_ace2      GGYMSGTSTLDVYDADIVVATSDIIVASMQYRVGSFGFLYLKPFFG-PD-SEEAPGNMGM
Api_mel_ace2      GGFMSGTATLDVYNADIMAATSNVIIASMQYRVGAFGFLYLNKHFT--N-SEEAPGNMGL
Bem_tab_ace2      GGYMTGTSTLELYDADIVAGVCNVIVASLQYRVGSFGFLYLKPLLP-EG-IEEAPGNMGL
Dros              GGFMTGSATLDIYNADIMAAVGNVIVASFQYRVGAFGFLHLAPEMPSEF-AEEAPGNVGL
Mus_dom           GGFMTGSATLDIYNAEIMSAVGNVIVASFQYRVGAFGFLHLSPVMP-GF-EEEAPGNVGL
Cte_fel_ace2      GGYMSGSAALDIYNAEILSSTGNVIVAAMQYRVGAFGFLYLAPHFR-NG-ASEAPGNMGL
Celeg_ace2        GGFFSGTPSLDLYSGSVFAAKEHTIVVNVNYRLGPFGFLYFGD-------DSPIQGNMGL
Cae_bri_ace2      GGFFSGTPSLDLYSGSVFAAKEHTIVVNVNYRLGPFGFLYFGD-------DSPIQGNMGL
```

```
                                              200
                                               ↓
Bla_ger_ace1      FDQLMALQWVHDNIQAFGGNPNNVTLFGESAGAVSVSLHLLSPLSRNLFSQAIMESGSPT
Nep_cin_ace1      FDQLMALQWVHDNIHFFGGNPHNVTLFGESAGAVSVSLHLLSPLSRNLFSQAIMESGSAT
Lip_ent_ace1      FDQLMALQWVHDNIHAFGGNPHNVTLFGESAGAVSVSTHLLSPLSRNLFSQAIMESGSPT
Cim_lec_ace1      YDQRMALQWVHDNIHLFGGDPQKVTLFGESAGAVSVSLHLLSPLSHKLFNQAIMESGSAV
Bom_man_ace1      FDQLMALQWVKDNIGYFGGNPHNITLFGESAGAVSVSLHLLSPLSRNLFSQAIMQSGAAT
Bom_Mor_ace1      FDQLMALQWVKDNIGYFGGNPHNITLFGESAGAVSVSLHLLSPLSRNLFSQAIMQSGAAT
Chi_sup_ace1      FDQLMALQWVKDNIAYFGGNPHNVTLFGESAGAVSVSLHLLSPLSRNLFSQAIMQSGAAT
Bem_tab_ace1      FDQLMALQWIRNNIHAFGGNPHNITLFGESAGAVSVSMHLLSPLSRNLFSQAIMESGSAT
Api_mel_ace1      FDQVMALEWVRDNIAAFGGNPDNVTLFGESAGAVSVSMHLLSPLSRHLFNQAIMQSGSPT
Cul_pip           FDQNLALRWVRDNIHRFGGDPSRVTLFGESAGAVSVSLHLLSALSRDLFQRAILQSGSPT
Cul_qui           FDQNLALRWVRDNIHRFGGDPSRVTLFGESAGAVSVSLHLLSALSRDLFQRAILQSGSPT
Aed_alb           FDQNLALRWVRDNIHKFGGDPSRVTLFGESAGAVSVSLHLLSALSRDLFQRAILQSGSPT
Ano_gam           FDQNLALRWVRDNIHRFGGDPSRVTLFGESAGAVSVSLHLLSALSRDLFQRAILQSGSPT
Cte_fel_ace1      FDQLLALRWVRDNVRAFGGDPDRVTLFGESAGAVSVSMHLLSPLSKDLFARAILESGSPT
LS_ace1-A         YDQMMALQWVKNNIEEFGGDPDKITI[FGESAG]GCSVALHLLSPLSRNLFSQAIMQSASAL
LS_ace1-B         YDQMMALQWVKNNIEAFGGDPDKITI[FGESAG]GCSVALHLLSPLSRNLFSQAIMQSSSAL
Tet_urt_ace1      FDQLMAMEWIRENIAAFGGNPANITIFGESAGAVSAALHLLSPLSRNVFSQAILQSGSAT
Rhi_dec           YDQYMALKWVQENVAAFGGDPDRVTLFGESAGAVSVGLHVLSPLSESLFHRVILQSGSPG
Celeg_ace1        WDQLLAMKWVHKNIDLFGGDLSRITLFGESAGAASVSIHMLSPKSAPYFHRAIIQSGSAT
Cae_bri_ace1      WDQLLAMKWVHKNIDLFGGDTSRITLFGESAGAASVSIHMLSQKSAPYFHRAIIQSGSAT
Homosap           LDQRLALQWVQENVAAFGGDPTSVTLFGESAGAASVGMHLLSPPSRGLFHRAVLQSGAPN
Tor_cal           LDQRMALQWVHDNIQFFGGDPKTVTIFGESAGGASVGMHILSPGSRDLFRRAILQSGSPN
Bom_man_ace2      WDQQLAIRWIKENARAFGGDPELITLFGESAGGGSVSLHMLSPEMKGLFKRGILQSGTLN
Bom_Mor_ace2      WDQQLAIRWIKENARAFGGDPELITLFGESAGGGSVSLHMLSPEMKGLFKRGILQSGTLN
Chi_sup_ace2      WDQQLAIRWIKENARAFGGDPELITLFGESAGGGSVSLHMLSPEMKGLFKRGILQSGTLN
Lep_dec           WDQILAIRWIKDNAAAFGGDPDLITLFGESAGGGSISIHLISPVTKGLVRRGIMQSGTMN
Nep_cin_ace2      WDQALAIQWIKANIANFGGDPELCTLFGESAGGGSVSLHLVSPVTRGLVRRGIMQSGTLN
Cim_lec_ace2      WDQAMAIRWIKDNIENFGGDPELITLFGESAGGGSVSIHLISPVTRGLARRGIMQSGTIN
Lip_ent_ace2      WDQAMAIRWLKDNAEAFGGDPELLTLFGESAGGGSVSLHLMSPVTKGLVRRGILQSGTLN
Bla_ger_ace2      WDQALAIRWLKDNAAAFGGDPDLITLFGESAGGGSVSLHLMSPITKGLVRRGIMQSGTLN
Api_mel_ace2      WDQALALRWLRDNAEAFGGDPELITIFGESAGGSSVSLHLISPVTRGLVRRGILQSGTLN
Bem_tab_ace2      WDQAMAIKWIKDNIAAFGGDPDMLTLFGESAGGSSVNIHLISPVTKGLARRGILQSGTLN
Dros              WDQALAIRWLKDNAHAFGGNPEWMTLFGESAGSSSVNAQLMSPVTRGLVKRGMMQSGTMN
Mus_dom           WDQALALRWLKENARAFGGNPEWMTLFGESAGSSSVNAQLMSPVTRGLVKRGMMQSGTMN
Cte_fel_ace2      WDQALAIRWLKDNAAAFGGDPERLTLFGESAGAGSVSLHLLSPATRGLFARALLQSGTIN
Celeg_ace2        MDQQLALRWVHENIGAFGGDRSRVTLFGESAGSASTTAHLFAPNSHKYFRNIIAKSGSII
Cae_bri_ace2      MDQQLALKWVHENIGAFGGDRSRVTLFGESAGSTSATAHLFAPNSHKYFRNIIAKSGSII
```

```
                   ●233                 254            265            ●279
Bla_ger_ace1      APWAIISREESILRGLRLAEAVGCPRSRS---DIRAVIDCLRKKNATDLVNNEWG----T
Nep_cin_ace1      APWAIISRDESFVRGLRLAEAVGCPHTRA---EIHEAIDCLRKKNASELVENEWG----T
Lip_ent_ace1      APWAIISREESILRGLRLAEAVNCPHDKN---QIKSVIECLRNTNASVLVDNEWG----T
Cim_lec_ace1      APWAIISREESMLRGLRLAEAVGCPHSKH---ELRAVIDCLRNTNATDLVSNEWG----T
Bom_man_ace1      APWAIISREESILRGIRLAEAVHCPHSRS---DLAPMIECLRKKNADELVNNEWG----T
Bom_Mor_ace1      APWAIISREESILRGIRLAEAVHCPHSRS---DLAPMIECLRKKNADELVNNEWG----T
Chi_sup_ace1      APWAIISREESILRGTRLAEAVHCPHSLK---DMGPMIECLRKKSADELVNNEWG----T
Bem_tab_ace1      APWAIISRQESIIRGLRLAEAVGCPHTRA---QIPEAIECLRKVNASVLVENESG----T
Api_mel_ace1      APWAIISREESIVRGIRLAEAVGCPHDRD---NLQEVIDCLRVKDPVELVKNEWG----T
Cul_pip           APWALVSREEATLRALRLAEAVNCPHDAT---KLSDAVECLRTKDPNELVDNEWG----T
Cul_qui           APWALVSREEATLRALRLAEAVNCPHDAT---KLSDAVECLRTKDPNELVDNEWG----T
Aed_alb           APWALVSREEATLRALRLAEAVNCPHDAS---KLTDTVECLRTKDPNVLVDNEWG----T
Ano_gam           APWALVSREEATLRALRLAEAVGCPHEPS---KLSDAVECLRGKDPHVLVNNEWG----T
Cte_fel_ace1      APWALRSRQEALNRSLLLAKTVGCPHSPD---DLAATAECLRQKDSRDLVNNEWN----D
LS_ace1-A         VPWGVITKKESIIRGRRLAEMMSCPYDEK---NTKAMIECLRQKDATEMVNQEWIGI--I
LS_ace1-B         VPWGVISKKESIRRGRRLAEEMRCPYGEN---NTNAMIECLLQKDATELVNQEWSGT--V
Tet_urt_ace1      CPWAISDRKKAYQRSLALAQAVGCGSTSTR--SVHAIIECMQSIPASELVAQEET----T
Rhi_dec           VPWGFQDRDKARQSAKRLATALRAPDSLD-----QETLDSLRCERPEDIVNNETN----S
Celeg_ace1        SPWAIEPRDVALARAVILYNAMKCGNMSLINPDYDRILDCFQRADADALRENEWAP---V
Cae_bri_ace1      SPWAIEPRDVALARAVILYNAMKCGNMSLISPDYDRILDCFQRADADALRENEWAP---V
Homosap           GPWATVGMGEARRRATQLAHLVGCPPGGT-GGNDTELVACLRTRPAQVLVNHEWHVL-PQ
Tor_cal           CPWASVSVAEGRRRAVELGRNLNCN-----LNSDEELIHCLREKKPQELIDVEWNVL-PF
Bom_man_ace2      APWSWMTGERAQDIGKVLIDDCNCNSSLLA-KDPSLVMDCMRGVDAKTISVQQWNS---Y
Bom_Mor_ace2      APWSWMTGERAQDIGKVLIDDCNCNSSLLA-KDPSLVMDCMRGVDAKTISVQQWNS---Y
Chi_sup_ace2      APWSWMTGERAQDIGKVLVDDCNCNSSLLT-ADPSLVMDCMRGVDAKTISVQQWNS---Y
Lep_dec           APWSYMSGERAEQIGKILIQDCGCNVSLLE-NSPRKVMDCMRAVDAKTISLQQWNS---Y
Nep_cin_ace2      APWSYMTGERAVEIAKTLIDDCGCNASMLI-ESPSRVMSCMRAVDAKTISVQQWNS---Y
Cim_lec_ace2      APWSFMTGERALEIGKILVEDCGCNVSQLA-ESPSRVMACLRAVDAKSISVHQWDS---Y
Lip_ent_ace2      APWSYMEAPKAVDIAKQLIDDCGCNSSILA-DFPHEVMTCMRNVEPKLISVQQWNS---Y
Bla_ger_ace2      APWSYMTGEKAADIGRVLVEDVGCNSTQLS-EAPSKVMACLRSVDSKAISVKQWNS---Y
Api_mel_ace2      APWSYMSGEKANEVATILVDDCGCNSTMLN-ENPARVMACMRSVDAKTISVQQWNS---Y
Bem_tab_ace2      MPWSYMEAEKAMQIGKILVDDCNCNSSQLE-ENPTKVFACMRAVDAKIVSSQQWSS---Y
Dros              APWSHMTSEKAVEIGKALINDCNCNASMLK-TNPAHVMSCMRSVDAKTISVQQWNS---Y
Mus_dom           APWSHMTSEKAVEIGKALVNDCNCNASLLP-ENPQAVMACMRQVDAKTISVQQWNS---Y
Cte_fel_ace2      APWSHMTAQDAVRVAEALVEDCGCNATLLR-DSPSMVLACMRSVDAKTISVQQWNS---Y
Celeg_ace2        NSWASATPPTMLDLSFRLAKKVNCSSPDMN-----AIVKCLRSVPAHLVQAEADNISGDI
Cae_bri_ace2      NSWASAPPPTMLDLSFRLAKKVNCSSTDMN-----VVAKCLRSVPAHLVQAEADNISGDI
```

```
                                                                                    327
                     288 ○ ● 290                                                    ↓  330 ● ● 331 ● 334
Bla_ger_ace1        LGICEFPFVPI-IDGTILDGPPQRSLAEKNFKK-TNILMGSNTEEGYYFIIYYLTELFRK
Nep_cin_ace1        LGICEFPFVPI-VDGAFLDDLPVRSLATKNFKK-TNILMGSNTEEGYYFIIYYLTELFRK
Lip_ent_ace1        LGICEFPFVPV-IDGSFLDETPQKSLANKNFKK-TNILMGSNTEEGYYFIIYYLTELLRK
Cim_lec_ace1        LGICEFPFVPI-VDGTFVDDHPKRNLAARNFKK-TNILMGSNTEEGYYFIIYYLTELFRK
Bom_man_ace1        LGICEFPFVPI-IDGSFLDEMPVRSLAHQNFKK-TNILMGSNTEEGYYFILYYLTELFPK
Bom_Mor_ace1        LGICEFPFVPI-IDGSFLDEMPVRSLAHQNFKK-TNILMGSNTEEGYYFILYYLTELFPK
Chi_sup_ace1        LGICEFPFVPI-IDGSFLDEMPIRSLAHQNFKK-TNILMGSNTEEGYYFILYYLTELFPK
Bem_tab_ace1        LGICDFPFVPV-VDGSFLDEMPSKSLATKNFKK-TNILMGSNTEEGNYFIMYYLTDLFRK
Api_mel_ace1        LGICEFPFVPV-IDGAFLDETPQRSLATSSFKK-ANIMMGSNTEEGFYFIIYYLTELFHI
Cul_pip             LGICEFPFVPV-VDGAFLDETPQRSLASGRFKK-TDILTGSNTEEGYYFIIYYLTELLRK
Cul_qui             LGICEFPFVPV-VDGAFLDETPQRSLASGRFKK-TDILTGSNTEEGYYFIIYYLTELLRK
Aed_alb             LGICEFPFVPV-VDGAFLDETPQRSLASGRFKK-TDILTGSNTEEGYYFIIYYLTELLRK
Ano_gam             LGICEFPFVPV-VDGAFLDETPQRSLASGRFKK-TEILTGSNTEEGYYFIIYYLTELLRK
Cte_fel_ace1        LGICEFPFVPV-VDGAFLDESPQRALKRGNFKK-TDILTGSNTEEGYYFIIYYLTELFKK
LS_ace1-A           SGIAEFPFVPI-VDGSFLDESPGKSLTTKNYKK-TNILIGANKEEGNYFIMYYLTDLFKN
LS_ace1-B           FGISEFPFVPI-VDGKFMDKTPEKSLKEKDYKK-TNILMGVNKDEGNFFIMYYLPELFKK
Tet_urt_ace1        TGVVEFAFIPI-VDGSFLDEDPEVSLRTKNFKH-TPILTGSNRDEGTYFLVYHSPHIFNL
Rhi_dec             GGVVDFPFVPV-VDGVFLPDTPQTLMDKGSFARNISVMLGSNANEGSWFLQYFFG--FPV
Celeg_ace1          REFGDFPWVPV-VDGDFLLENAQTSLKQGNFKK-TQLLAGSNRDESIYFLTYQLPDIFPV
Cae_bri_ace1        REFGDFPWVPV-VDGDFLLENAQTSLKQGNFKK-TQLLAGSNRDESIYFLTYQLPDIFPV
Homosap             ESVFRFSFVPV-VDGDFLSDTPEALINAGDFHG-LQVLVGVVKDEGSYFLVYGAPGFSKD
Tor_cal             DSIFRFSFVPV-IDGEFFPTSLESMLNSGNFKK-TQILLGVNKDEGSFFLLYGAPGFSKD
Bom_man_ace2        TGILGFPSAPT-VDGIFLPKDPDTMMKEGNFHN-SEVLLGSNQDEGTYFLLYDFLDYFEK
Bom_Mor_ace2        TGILGFPSAPT-VDGIFLPKDPDTMMKEGNFHN-SEVLLGSNQDEGTYFLLYDFLDYFEK
Chi_sup_ace2        TGILGFPSAPT-VDGVFLPKDPDTMMKEGNFHN-TEVLLGSNQDEGTYFLLYDFLDYFEK
Lep_dec             SGILGFPSTPT-IEGVLLPKHPMDMLAEGDYED-MEILLGSNHDEGTYFLLYDFIDFFEK
Nep_cin_ace2        FGILGFPSAPT-IDGVFLPKHPLDLLKEGDFQD-TEILIGSNQDEGTYFILYDFIDYFEK
Cim_lec_ace2        FSILNFPSAPT-IDGNFLPKHPLELLAEGDFPE-TEIIIGSNLDEGTYFMLYDFIDYFEK
Lip_ent_ace2        WGILGFPSAPT-IDGVFLPEHRLALLKKGDFPE-TEIMIGSNLDEGTYFILYDFIDYFEK
Bla_ger_ace2        WGILGFPSAPT-IDGVFLPKHPMDLIKELDWED-TEILIGSNQDEGTYFILYDFMDYFEK
Api_mel_ace2        WGILGFPSAPT-IDGIFLPKHPLDLLREADFKD-TEILIGNNENEGTYFILYDFNDIFEK
Bem_tab_ace2        FGILGYPSAPT-IDGEFLPKHPLELMKDQNFED-IELLIGSNRDEGTYFLLYDFLEFFEK
Dros                SGILSFPSAPT-IDGAFLPADPMTLMKTADLKD-YDILMGNVRDEGTYFLLYDFIDYFDK
Mus_dom             SGILSFPSAPT-IDGAFLPADPMTLLKTADLSG-YDILIGNVKDEGTYFLLYDFIDYFDK
Cte_fel_ace2        SGILGFPSAPT-IDGVFMTGDPMQMLRNADLQG-VDVMIGSNKDEGTYFILYDFIDYFEK
Celeg_ace2          GPPMTFAYVPVSSDANFFQGDVFQKLANKQFKKDVNIIFGSVKDEGTYWLPYYMSLPKYG
Cae_bri_ace2        GPPMTFAYVPVSSDANFFQGDVIQKLNNKQFKKDVNIIFGSVKDEGTYWLPYYMSLPKYG
```

```
Bla_ger_ace1      E------------ENVYVNREEFLRSVQELN---PYVNNVARQAIVFEYTDWLNPDDPIR
Nep_cin_ace1      E------------ENVYVBRDEFLHAVHELN---PYVNNVARQAIVFEYTDWLNPDDPIR
Lip_ent_ace1      E------------ENVYVNRDEFLQAVRELN---PYINKVARQAIIFEYTDWLNPDDPVR
Cim_lec_ace1      E------------ENVYINREEFLRAVVELN---PYVNNIARQAIIFEYTDWQNPEDPIK
Bom_man_ace1      E------------ENVGISREQFLQAVRELN---PYVNDVARQAIIYEYTDWLNPEDPVK
Bom_Mor_ace1      E------------ENVGISREQFLQAVRELN---PYVNDVARQAIIYEYTDWLNPEDPVK
Chi_sup_ace1      E------------ENVGITREQYLQAVRELN---PYVSDVGRQAIVFEYTDWLNPEDPVR
Bem_tab_ace1      E------------ENIHVSRDQFIQAVSELN---PYN-FIVRRAIIFEYTDWLNPDDPVK
Api_mel_ace1      DG-----------SEVKVSREQFISAVSELN---PYVNQFGRRAIIYEYTDWLRPDDPHA
Cul_pip           E------------EGVTVTREEFLQAVRELN---PYVNGAARQAIVFEYTDWIEPDNPNS
Cul_qui           E------------EGVTVTREEFLQAVRELN---PYVNGAARQAIVFEYTDWIEPDNPNS
Aed_alb           E------------EGVTVSREEFLQAVRELN---PYVNGAARQAIVFEYTDWTEPENPNS
Ano_gam           E------------EGVTVTREEFLQAVRELN---PYVNGAARQAIVFEYTDWTEPDNPNS
Cte_fel_ace1      E------------EGINVTREQFLQAVKDLN---PRVGPIGTQAIVFEYTDWLDPEDPLG
LS_ace1-A         T------------ESVYVDRTDFIRSVSELN---HYVKKMGREAITFEYTDWLNPNDPIK
LS_ace1-B         N------------ENVYINRTDFIRSVSDLN---IYVNNAGREAITFEYTDWLNPNDPIK
Tet_urt_ace1      S------------EGIYISRSEFQSLIRIIY---PHLSPLAQEAVIQEYTHWINPDDQIE
Rhi_dec           SD-----------ETPEVTKENFTAVLEALD---PSLEHTPIAEIMKTYTAGEIPSTAAD
Celeg_ace1        ADF-------FTKTDFIKDRQLWIKGVKDLLPRQILKCQLTLAAVLHEYEPQDLPVTPRD
Cae_bri_ace1      ADF-------FSKSEFIKDRQTWIKGVKDLLPRQILKCQLTLAAVLHEYEPQDLPISAQN
Homosap           N-------------ESLISRAEFLAGVRVGV---PQVSDLAAEAVVLHYTDWLHPEDPAR
Tor_cal           S-------------ESKISREDFMSGVKLSV---PHANDLGLDAVTLQYTDWMDDNNGIK
Bom_man_ace2      D------------GPSFLQREKFLEIVDTIFK---DFSKIKREAIVFQYTDWEEITDGYL
Bom_Mor_ace2      D------------GPSFLQREKFLEIVDTIFK---DFSKIKREAIVFQYTDWEEITDGYL
Chi_sup_ace2      D------------GPSFLQREKFLEIVDTIFK---EFSKIKREAIVFQYTDWEEITDGYL
Lep_dec           D------------GPSFLQREKYHDIIDTIFK---NMSRLERDAIVFQYTNWEHVHDGYL
Nep_cin_ace2      D------------GPSFLQRDKFLDIINTIFK---NFTRLERDAIIFQYTDWEHANDGYL
Cim_lec_ace2      D------------GPIFLQRDKYLDIVNTIFK---NMITLERDAIIFQYTDWEKVNDEHL
Lip_ent_ace2      D------------GPSFLQRDKFLEIINTIFK---NFSRIEREAIVFQYTDWDQSNDGFL
Bla_ger_ace2      D------------SPTFLQRDKYLDIVNLIFK---NMTRLERDAIIFQYTDWEHLADGYK
Api_mel_ace2      D------------QASFLERERFLGIINNIFK---NMSQIEREAITFQYTDWEEVYNGYI
Bem_tab_ace2      D------------GPSLLQRDKFLDIIHTIFK---NFSPLEKEAIIFQYTDWENLGDGYT
Dros              D------------DATALPRDKYLEIMNNIFG---KATQAEREAIIFQYTSWE-GNPGYQ
Mus_dom           D------------DATSLPRDKYLEIMNNIFQ---KASQAEREAIIFQYTSWE-GNPGYQ
Cte_fel_ace2      D------------GPSILQRDKFLEIMSTIFV---KASPAERQAIDFQYTDWENPTDGSL
Celeg_ace2        FAFNHTISAEDPHNRALITRDHYEESMRAFMPYFAGSKLVLNAFMNSYEHVSTSNVPEER
Cae_bri_ace2      FAFNHTISAEDPHNRALITREHYEESMKAFMPYFAGSKLVLNAFMNSYEHVSTSNVPEER
```

402 432 440 442

```
Bla_ger_ace1     NRDALDKMVGDYHFTCNVNEFAHRYAETGN-NVYMYYFKHRSVGNPWPSWTGVMHGDEIN
Nep_cin_ace1     NRDALDKMVGDYHFTCNVNEFAHRYAETGN-NVYMYYFKYRSIGNPWPSWTGVMHADEIN
Lip_ent_ace1     SRDALDKMVGDYHFTCNVNEFAHRYAETGN-NVYMYYFKHRSAANPWPSWTGVMHGDEIN
Cim_lec_ace1     NRDALDKMVGDYQFTCTVNEFAHTYAESGN-VVYMYLFSHRSIGNPWPSWTGVMHGDEIN
Bom_man_ace1     NRNALDKMVGDYHFTCGVNEFAHRYAETGN-NVYTYYYKHRSKNNPWPSWTGVMHADEIN
Bom_Mor_ace1     NRNALDKMVGDYHFTCGVNEFAHRYAETGN-NVYTYYYKHRSKNNPWPSWTGVMHADEIN
Chi_sup_ace1     NRNALDKMVGDYHFTCGVNEFAHRYAETGN-NVYTYYYKHRSKNNPWPSWTGVMHADEIN
Bem_tab_ace1     NRDALDKIVGDFHFTCNVNEFAYRYAETGN-TVYMYYFKHRSTSSPWPTWSGALHGDEIN
Api_mel_ace1     NRDALDKIVGDYQFTCNVNEFAGRYTDTGN-TVYMYYYKHRSMNNPWPRWTGVMHADEIS
Cul_pip          NRDALDKMVGDYHFTCNVNEFAQRYAEEGN-NVFMYLYTHRSKGNPWPRWTGVMHGDEIN
Cul_qui          NRDALDKMVGDYHFTCNVNEFAQRYAEEGN-NVFMYLYTHRSKGNPWPRWTGVMHGDEIN
Aed_alb          NRDALDKMVGDYHFTCNVNEFAQRYAEEGN-NVYMYLYTHRSKGNPWPRWTGVMHGDEIN
Ano_gam          NRDALDKMVGDYHFTCNVNEFAQRYAEEGN-NVYMYLYTHRSKGNPWPRWTGVMHGDEIN
Cte_fel_ace1     NRDALDKIVGDYQFTCNVNEFAHRYASEGL-NVYMYLFSHRSRINPWPRWTGVMHGDEIS
LS_ace1-A        NREAIDRMVGDYQFICPTADFARIYASTGN-NVYMYYFTERSSTSPWPTWSGVLHGDEIA
LS_ace1-B        NREAIDRMVGDYQFTCPTADFARIYASTGN-NIYMYYFTERSSTSPWPTWSGVLHGDEIA
Tet_urt_ace1     NREATDKFVGDYHFTCPVNEMSYRYALYGN-DVWTYHFTHRSSKSFWPSWMGVIHGEEIK
Rhi_dec          ILKALDSIVGDYHFTCPAVRWADTFARAGI-PVYQYVFARRSSRNPWPQWTGVMHGEEVP
Celeg_ace1       WINAMDKMLGDYHFTCSVNEMALAHTKHGG-DTYYYYFTHRASQQTWPEWMGVLHGYEIN
Cae_bri_ace1     WLNAMDKMLGDYHFTCSVNEMALAHTKHGG-DTFYYYFTHRATQQTWPEWMGVLHGYEIN
Homosap          LREALSDVVGDHNVVCPVAQLAGRLAAQGA-RVYAYVFEHRASTLSWPLWMGVPHGYEIE
Tor_cal          NRDGLDDIVGDHNVICPLMHFVNKYTKFGN-GTYLYFFNHRASNLVWPEWMGVIHGYEIE
Bom_man_ace2     NQKMIADVVGDYFFVCPTNYFAEILADAGV-DVYYYYFTHRTSTSLWGEWMGVMHGDEME
Bom_Mor_ace2     NQKMIADVVGDYFFVCPTNYFAEILADAGV-GVYYYYFTHRTSTSLWGEWMGVMHGDEME
Chi_sup_ace2     NQKMIADVVGDYFFVCPTNYFAEILADAGV-DVYYYYFTHRTSTSLWGEWMGVMHGDEME
Lep_dec          NQKMIGDVVGDYFFVCPTNNFAEVAADRGM-KVFYYYFTHRTSTSLWGEWMGVIHGDEVE
Nep_cin_ace2     NQKMIGDVVGDYFFICPTNLFAQAFADHGL-KVFYYFFTQRTSTSLWGEWMGVMHGDEIE
Cim_lec_ace2     NQKMVGDIIGDYFFICPTNQFAQMFAEHGL-KVYYYFFTQRPSTSPWGEWMGVMHGDEVG
Lip_ent_ace2     NQKMIADVGGDYYFVCPSNLFAEMFADVGM-KVYYYYFTQRTSTDLWGEWMGVMHGDEIE
Bla_ger_ace2     NQRMIADVVGDYFFICPTNVFAEQFAEHGT-KVYYYYFTQRTSLNLWGQWMGVMHGDEIE
Api_mel_ace2     YQKMVADVVGDYFFICPSIHFAQLFADRGM-KVYYYFFTQRTSTNLWGEWMGVLHGDEVE
Bem_tab_ace2     NQKMIGEIVGDYFFICPSNYFAQVMSDNGA-KIYYYYFTQRTSTNLWGEWMGVMHGDEVE
Dros             NQQQIGRAVGDHFFTCPTNEYAQALAERGA-SVHYYYFTHRTSTSLWGEWMGVLHGDEIE
Mus_dom          NQQQIGRAVGDHFFTCPTNEYAQALAERGA-SVHYYYFTHRTSTSLWGEWMGVLHGDEIE
Cte_fel_ace2     NQDQVGRAVGDHFFVCPSNLFAEGLAERGA-NVRYYYFTHRTSTSVWGEWMGVMHGDEIE
Celeg_ace2       YRDGVARFLGDLFFTCSLIDFADLISDNIFGNVYMYYFTYRSSANPWPKWMGVMHGYEIE
Cae_bri_ace2     YRDGVARFLGDLFFTCSLIDFADLISDNIFGNVYMYYFTYRSSANPWPRWMGVMHGYEIE
```

```
Bla_ger_ace1      YVFGEPLNPAKN----YQPQEIELSRRMMRYWANFAKTGNP--SMSEDGTWTATYWPVHT
Nep_cin_ace1      YIFGEPLNPILN----YHPQEVELSRRMMRYWANFAKTGNP--SMSEDGTWTATYWPVHT
Lip_ent_ace1      YVFGEPLNPKKN----YQPQEKILSKRMMRYWANFAKTGNP--SMSEDGTWTDVYWPVHT
Cim_lec_ace1      YVFGEPLNPTKN----YLPSEAELSRRMMNYWANFAKTGNP--NLKGNNSWTSTYWPQHT
Bom_man_ace1      YVFGEPLNPGKN----YSPEEVEFSKRLMRYWANFARSGNP--SLNPNGEMTKIHWPVHT
Bom_Mor_ace1      YVFGEPLNPGKN----YSPEEVEFSKRLMRYWANFARSGNP--SLNPNGEMTKIHWPVHT
Chi_sup_ace1      YVFGEPLNPGKN----YSPEEVEFSKRIMRYWANFARSGNP--SLNPNGDMTKVHWPVHT
Bem_tab_ace1      YIFGEPLNPTKK----YQPAEVELAKRMMRYWANFAKTGNP--SLSSDGSWASVYWPQHT
Api_mel_ace1      YVFGEPLDPTKG----YTPEEVNLSKKMMRYWANFAKTGDP--NVGDVDVWTQAYWPPHT
Cul_pip           YVFGEPLNSALG----YQDDEKDFSRKIMRYWSNFAKTGNP--NPSTPSVDL-PEWPKHT
Cul_qui           YVFGEPLNSALG----YQDDEKDFSRKIMRYWSNFAKTGNP--NPSTPSVDL-PEWPKHT
Aed_alb           YVFGEPLNSDLG----YMEDEKDFSRKIMRYWSNFAKTGNP--NPSPPNSDF-TEWPKHT
Ano_gam           YVFGEPLNPTLG----YTEDEKDFSRKIMRYWSNFAKTGNP--NPNTASSEF-PEWPKHT
Cte_fel_ace1      YVFGEPQDSSRG----YTHAEAALSKRMMRYWANFAKTGDP--NPG-PGNEP-IYWPKHT
LS_ace1-A         FVFGEALNKSKN----YDKSEIALSKRMMGYWANFAKTGNP--SLSADGTWSTNYWPLHT
LS_ace1-B         FVFGEPLNTSKN----YDDSEIALSKRIMSYWANFAKTGNP--NVLANGNYSNKIWPLHT
Tet_urt_ace1      FVLGEPLDPVHG----YTPAEVQLSKRIMRYWANFARTGNPNKQFPDGDDTESIVWPEYT
Rhi_dec           FVFGEPLNDTHC----YSEEDKTLSRRIMRYWANFAKTGNPN-LTEDGSFGSTIHWPERT
Celeg_ace1        FIFGEPLNQKRFN---YTDEERELSNRFMRYWANFAKTGDP--NKNEDGSFTQDVWPKYN
Cae_bri_ace1      FIFGEPFNQKRFN---YTDEERELSNRFMRYWANFAKTGDP--NKNEDGSFTQDIWPKYN
Homosap           FIFGIPLDPSRN----YTAEEKIFAQRLMRYWANFARTGDP----NEPRDPKAPQWPPYT
Tor_cal           FVFGLPLVKELN----YTAEEEALSRRIMHYWATFAKTGNP----NEPHSQES-KWPLFT
Bom_man_ace2      YVFGHPLNMSLQ----YHSRERDLAAHIMQSFTQFALTGKP--------HKPDEKWPLYS
Bom_Mor_ace2      YVFGHPLNMSLQ----YHSRERDLAAHIMQSFTQFALTGKP--------HEPDEKWPLYS
Chi_sup_ace2      YVFGHPLNMSLQ----YHTRERDLAAHIMQSFTRFALTGKP--------HKPDEKWPLYS
Lep_dec           YVFGHPLNMSLQ----FNSRERELSLKIMQAFARFATTGKP--------VTDDVNWPLYT
Nep_cin_ace2      YVFGHPLNMSLQ----YNARERDLSLRIMQAYSRFALTGKP--------VSDDINWPIYS
Cim_lec_ace2      YVFGRPLNMSLS----YSARERDLSLRIIEAFSTFALTGKP--------VPEDVNWPPYT
Lip_ent_ace2      YVFGHPLNMSIQ----YNKKERALSKRIMDTFTRFALTGKP--------MPEEREWPPYT
Bla_ger_ace2      YVFGHPLNMSID----YNDNERDLSLRIMEIYSRFALTGKP--------IANEADWPTYT
Api_mel_ace2      YVFGHPLNKSLK----YSDKERDLSLRMILYFSEFAYLGKP--------TKEDSEWPSYS
Bem_tab_ace2      YVFGHPLNMSLQ----YNARERDLSNRIMEAFSKFAMTGKP--------TGEDVTWPQYT
Dros              YFFGQPLNNSLQ----YRPVERELGKRMLSAVIEFAKTGNP--------AQDGEEWPNFS
Mus_dom           YFFGQPLNNSLQ----YRPVERELGKRMLNSVIEFAKSGNP--------AVDGEEWPNFS
Cte_fel_ace2      YIFGQPLNESLQ----YRDRERELSAMMVQSVADFARTGDP--------TPAGETWPLYS
Celeg_ace2        YAFGQPYWRPHLYDQTHLEDEKRLSSIIMQIWANFANTGRT-----------DSFWPQYN
Cae_bri_ace2      YAFGQPYWRPHLYDQKQLEDEKRLSSIIMQVWANFANTGRT-----------DSFWPQYN
```

521

```
Bla_ger_ace1      AYGREYLTLDVN----------------STETGRGPRLKQCAFWKKYLPQLIAVTSNLNQ
Nep_cin_ace1      AYGREYLTLDVN----------------STATGRGPRLKQCAFWKKYLPQLIAATEKLQA
Lip_ent_ace1      PFGREFLTLAVN----------------NTSTGRGPRLKQCAFWKKYLPQLVAVTANLNS
Cim_lec_ace1      AYGREFLTLGINQ--------------SSSTTGRGPRVKQCAFWKKYLPQLIASTETRTN
Bom_man_ace1      AFGREYLSLAVN----------------SSSIGRGLRVKQCAFWQKHLPQLMAATNKPEP
Bom_Mor_ace1      AFGREYLSLAVN----------------SSSVGRGLRVKQCAFWQKHLPQLMAATNKPEP
Chi_sup_ace1      AFGGEYLSLAVN----------------SSAVGHGLKVKQCAFWQKYLPQLMAATTKPEP
Bem_tab_ace1      TYGREFLTLDIN----------------STAIGQGPRLRQCAFWKKYLPQLISTTSYLVP
Api_mel_ace1      AAKKEYMTLDTN----------------SSEIGNGPRVRQCIFWKNYLPQLVAGTSKLEP
Cul_pip           AHGRHYLELGLN----------------TTFVGRGPRLRQCAFWKKYLPQLVAATSNLQV
Cul_qui           AHGRHYLELGLN----------------TTFVGRGPRLRQCAFWKKYLPQLVAATSNLQV
Aed_alb           AHGRHYLELGLN----------------TTYVGRGPRLRQCAFWKKYLPQLVAATSNLQA
Ano_gam           AHGRHYLELGLN----------------TSFVGRGPRLRQCAFWKKYLPQLVAATSNLPG
Cte_fel_ace1      VEHKEYLTLGVN----------------DSELGKGPRLRQCAFWKQFLPQLIIATKNTTA
LS_ace1-A         PTKQEVLELNAN----------------YSRVLEGLRVKKCAFWKKYLPKLLSLTSNNGD
LS_ace1-B         PIKQEVLELNAN----------------YSRVFEGLRVRKCAFWKTYLPKLLSLTSNNTK
Tet_urt_ace1      AHEKEYLVISTN----------------DSSIGRGLRAKQCAFWKNFLPKLINALENRHN
Rhi_dec           DSLKRHLVLDVN-----------------ESVGWAHRQTYCDFWENVRRNRTPPVPSC--
Celeg_ace1        SVSMEYMNMTVESS-----------YPSMKRIGHGPRRKECAFWKAYLPNLMAAVADVGD
Cae_bri_ace1      SVSMEYMNMTVESS-----------YPGQNRIGHGPRRKECAFWKAYLPNLMAAVADVGD
Homosap           AGAQQYVSLDLRP----------------LEVRRGLRAQACAFWNRFLPKLLSATDTLDE
Tor_cal           TKEQKFIDLNTEP----------------MKVHQRLRVQMCVFWNQFLPKLLNATACDGE
Bom_man_ace2      RSSPHYYTYTAV-------------GPSGPAGPRGPRASACAFWNDFLNKLN---ELERV
Bom_Mor_ace2      RSSPHYYTYTAV-------------GPSGPAGPRGPRASACAFWNDFLNKLN---ELERA
Chi_sup_ace2      RSSPHYYTYTAD-------------GTSGPAGPRGPRASACAFWNDFLNKLN---ELEHV
Lep_dec           KDQPQYFIFNAD-------------KNG---IGKGPRATACAFWNDFLPKLRDNSGSEEA
Nep_cin_ace2      REQPQYYIFNAE-------------KSG---IGKGPRATACAFWNEFLPRLRGQPDPECL
Cim_lec_ace2      KEQPYYYVFNAE-------------DMG---IGHGPRSTACAFWNDFFPKLKETPGENCG
Lip_ent_ace2      KEEPKYYIYNAE-------------SMG---TGKGPRSNPCAFWNDFLPKLQGNPRFEDQ
Bla_ger_ace2      KDHPKYYILNSD-------------TYG---TGKGPRTTNCAFWNDFLPKLANHPDPDYA
Api_mel_ace2      RDEPKYFIFDAE-------------KTG---LGKGPRTTYCAFWNEFLPKLKGIPDPXPN
Bem_tab_ace2      RSNPQYFIFHAT-------------TSG---LGSGPRLTACQFWNEFLPKLRNVSENISS
Dros              KEDPVYYIFST--------------DDKIEKLARGPLAARCSFWNDYLPKVRSWAGTCDG
Mus_dom           KEDPVYYVFST--------------DEKIEKLQRGPLAKRCSFWNDYLPKVRSWIGSECE
Cte_fel_ace2      REKPIYYEFNAEGPQPEPSLDIDGSDSDLEHTGRGPRATACAFWNEFLPKLRALSETPPS
Celeg_ace2        KIERKAIELGETTLQG------------KHRIISDVHGGFCRMIDEAKAFVKQKNANDCR
Cae_bri_ace2      KIERKAIELGESTLHG------------NHRIIADVHGSYCRMIDEAKAFVKQKNASDCR
```

```
Bla_ger_ace1      QPESCPTDTSGAEMNKESSLLLICLVMKIILLWGPRGWV---------------------
Nep_cin_ace1      PVVETCTGAAGEARVAWSLIAMAFALCVV-------------------------------
Lip_ent_ace1      NNPQPCASSSHKTFDVISFHVFTLIIISKLTHLWILQ-----------------------
Cim_lec_ace1      PVQECTNGVNSLSLSKELLPAAVAFFAALTVYV---------------------------
Bom_man_ace1      PKNCTNSVSSLWPSRKALSFNVIATAALTGTSLFKYTI----------------------
Bom_Mor_ace1      PKNCTNSVPSLWPSRNTLGFNVIATAALTGTALFKYTI----------------------
Chi_sup_ace1      IQNCTSNSGTRHYYGVTSLSLVTVFGFLQPTILKYIII----------------------
Bem_tab_ace1      ANSSNCTSAAPSSLLQHGFNKLSSHSSPFILLLAVFQFLLLLLSNSAIL-----------
Api_mel_ace1      KETCSGTRVNDGGRLLLVLSLVVIGTLFSHRIATRAYDELPEIFDPKGFV----------
Cul_pip           TPAPSVPCESSSTSYRSTLLLIVTLLLVTRFKI---------------------------
Cul_qui           TPAPSVPCESSSTSYRSTLLLIVTLLLVTRFKI---------------------------
Aed_alb           TPAPSEPCGSSAALYRPLLFLIVSLVLVTRFKI---------------------------
Ano_gam           PAPPSEPCESSAFFYRPDLIVLLVSLLTATVRFIQ-------------------------
Cte_fel_ace1      SVRSECVSGVPAPSACGAVIALFLLLCAILHRSPL-------------------------
LS_ace1-A         HQCQTSSTSSPSCCKDGTCCNSGTSFASTFFSVFIVTTSVHLFYNFKFSVIYSNINNGKL
LS_ace1-B         SEVVTNPS----------------------------------------------------
Tet_urt_ace1      STCTSHSNQIGSSNWSLAISLISLIMCFLPSLR---------------------------
Rhi_dec           ------------------------------------------------------------
Celeg_ace1        PYLVWKQQMDKWQNEYITDWQYHFEQYKRYQTYRQSDSETCGG-----------------
Cae_bri_ace1      PYLVWKQQMDKWQNEYITDWQYHFEQYKRYQTYRQSDSETCGG-----------------
Homosap           AERQWKAEFHRWSSYMVHWKNQFDHYSKQDRCSDL-------------------------
Tor_cal           LSS---------------------------------------------------------
Bom_man_ace2      PCDGAVTGPYSSVAGTALPVTLLTTLAITIAL----------------------------
Bom_Mor_ace2      PCDGAVTGPYSSVAGTALPVTLLTTLAITIAL----------------------------
Chi_sup_ace2      PCDRAVTGPYSSVAGTTLPILLLTALATSVAL----------------------------
Lep_dec           PCVNTYLSKIRSSSNELLPPSTSLVLIWIMTLLNAL------------------------
Nep_cin_ace2      ADVAEVETSSPLVDNVSDNSTSTTFKPCTVITVLGLLLLTI-------------------
Cim_lec_ace2      CDKSEPILDVELVQNMSMGIMLNRGNSESPWISLVFGLIVALAAV---------------
Lip_ent_ace2      GCNGKAEETFSNPMSRSSTLSKGIWLILLSSLAVILSL----------------------
Bla_ger_ace2      CASGVAETANLGVNSATSCLLVGPKHLLYFVTIVLARLLLSL------------------
Api_mel_ace2      TCKVIASSVSAGEEGLGNSLAITALSSILVLSRVI-------------------------
Bem_tab_ace2      TTDIKSCCSQEMSLGDCNNTALRVSDSGGRAAPEMGFLLLALFPVLLTSIR---------
Dros              DSGSASISPRLQLLGIAALIYICAALRTKRVF----------------------------
Mus_dom           NKSSTSASAAIYEMKMQQLTLLAVAIILTMVNSIFQ------------------------
Cte_fel_ace2      PCEITERIIQIASGCVNFKYLWTWSLLLNLMVINLV------------------------
Celeg_ace2        TTRKSASTEDLTSSSSTTYLFSIIVYLSILISYISL------------------------
Cae_bri_ace2      STRKSDSSSTETSSTANSLYLFIIISLSLLISCISL------------------------
```

Figure S1. Alignment of LS-ace1-A and LS-ace1-B proteins with other typical AChE proteins from other species (Insects, Nematods, Arachnida and vertebrates). By convention, numbering is that of *Torpedo californica*. The three amino acids composing the catalytic triad (S200, E327 and H440) are indicated by arrows. The 14 conserved aromatic residues lining the active gorge are represented by circles. Out of these 14, 11 residues were present in both LS-ace1-A and LS-ace-B (shown by filled circles), whereas the other 3 non conserved residues (shown by open circles) were absent in both the proteins of sea lice. The choline binding site (W at 84) is underlined. Three interchain disulphide bridges are drawn between conserved Cys residues (shown by arrows). The solid box represents the canonical *FGESAG* motif, characteristic of the active site of cholinesterases. The dotted box represents the typical sequence insertion/deletion domain that easily distinguishes ace 1 and ace 2 proteins.

Abbrevations: Insects: *Liposcelis entomophila* ace1: ACI16651.2 (Lip_ent_ace1), Liposcelis entomophila ace2: ACI16652.1 (Lip_ent_ace2), *Bemisia tabaci* ace1: ABV45413.1 (Bem_tab_ace1), *Bemisia tabaci* ace2 : ABV45415.1 (Bem_tab_ace2), *Blattella germanica* ace 1 : ABB89946.1 (Bla_ger_ace1), *Blattella germanica* ace 2 : ABB89947.1 (Bla_ger_ace2), *Nephotettix cincticeps* ace1 : BAI63797.1 (Nep_cin_ace1), *Nephotettix cincticeps* ace2 : BAI63643.1 (Nep_cin_ace2), *Ctenocephalides felis* ace1: CBI83253.1 (Cte_fel_ace1), *Ctenocephalides felis* ace2 : CBI83254.1 (Cte_fel_ace2), *Culex pipiens* ace : CAD33707.2 (Cul_pip), *Chilo suppressalis* ace1: EF453724 (Chi_sup_ace1), *Chilo suppressalis* ace2: EF470245 (Chi_sup_ace2), *Apis mellifera* ace1: XP_39375.1 (Api_mel_ace1), *Apis mellifera* ace2: NM_001040230.1 (Api_mel_ace2), *Cimex lectularius* ace1: JN563927.1 (Cim_lec_ace1), *Cimex lectularius* ace2: GU597839.1 (Cim_lec_ace2), *Bombyx mandarina* ace1: FJ542315.1 (Bom_man_ace1), *Bombyx mandarina* ace2: FJ542316.1 (Bom_man_ace2), *Bombyx mori* ace1: DQ186606.1 (Bom_Mor_ace1), *Bombyx mori* ace2: NM_001114641.1 (Bom_Mor_ace2), *Leptinotarsa decemlineata*: AAB00466.1 (Lep_dec), *Drosophila melanogaster*: XO5893 (Dros), *Musca domestica*: CAC39209.1 (Mus_dom), *Anopheles gambiae*: CAD56157.2 (Ano_gam), *Aedes albopictus*: BAE71348.1 (Aed_alb), *Culex quinquefasciatus*:

XP_001847448.1 (Cul_qui), Nematods: *Caenorhabditis elegans* ace1: CCD68912.1 (Celeg_ace1), *Caenorhabditis elegans* ace2: AF025378.3 (Celeg_ace2), *Caenorhabditis briggsae* ace1: AAB41269.1 (Cae_bri_ace1), *Caenorhabditis briggsae* ace2: AF030037.2 (Cae_bri_ace2), Arachnida: *Tetranychus urticae* ace1: ADK12687.1 (Tet_urt_ace1), *Rhipicephalus decoloratus*: CAA06980.1 (Rhi_dec), Vertebrates: *Torpedo californica*: CAA27169.1 (Tor_cal), *Homo sapiens*: AAA68151.1 (Homosap).
