# Supplementary figures and images for: Identification and Molecular Characterization of Two Acetylcholinesterases from the Salmon Louse, Lepeophtheirus salmonis
Source: PLoS One. 2015 May 4;10(5):e0125362. doi: 10.1371/journal.pone.0125362 (PMC4418574; doi:10.1371/journal.pone.0125362)

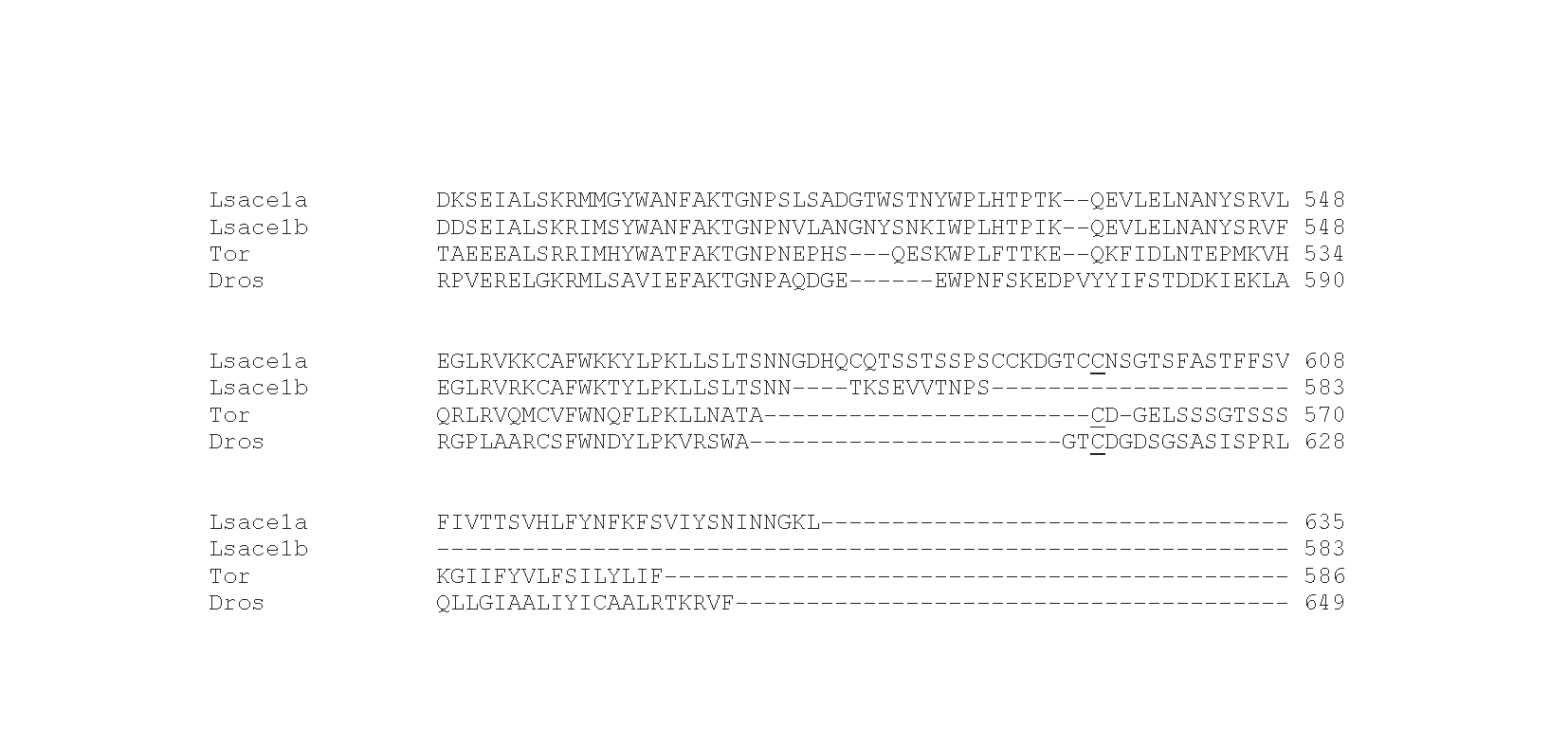

Supplement: S2 Fig — Alignment of Ls AChE1a and Ls AChE1b proteins with Torpedo californica AChE and Drosophila melanogaster AChE. The alignment corresponding to hydrophobic peptide sequences was manually edited. The site of cleavage (cysteine) of the hydrophobic peptide has been underlined in Ls AChE1a, Torpedo californica AChE and Drosophila melanogaster AChE. Ls AChE1b did not have the free cysteine residue that could serve as the site of cleavage of the hydrophobic peptide. (TIFF) [file pone.0125362.s002.tiff]

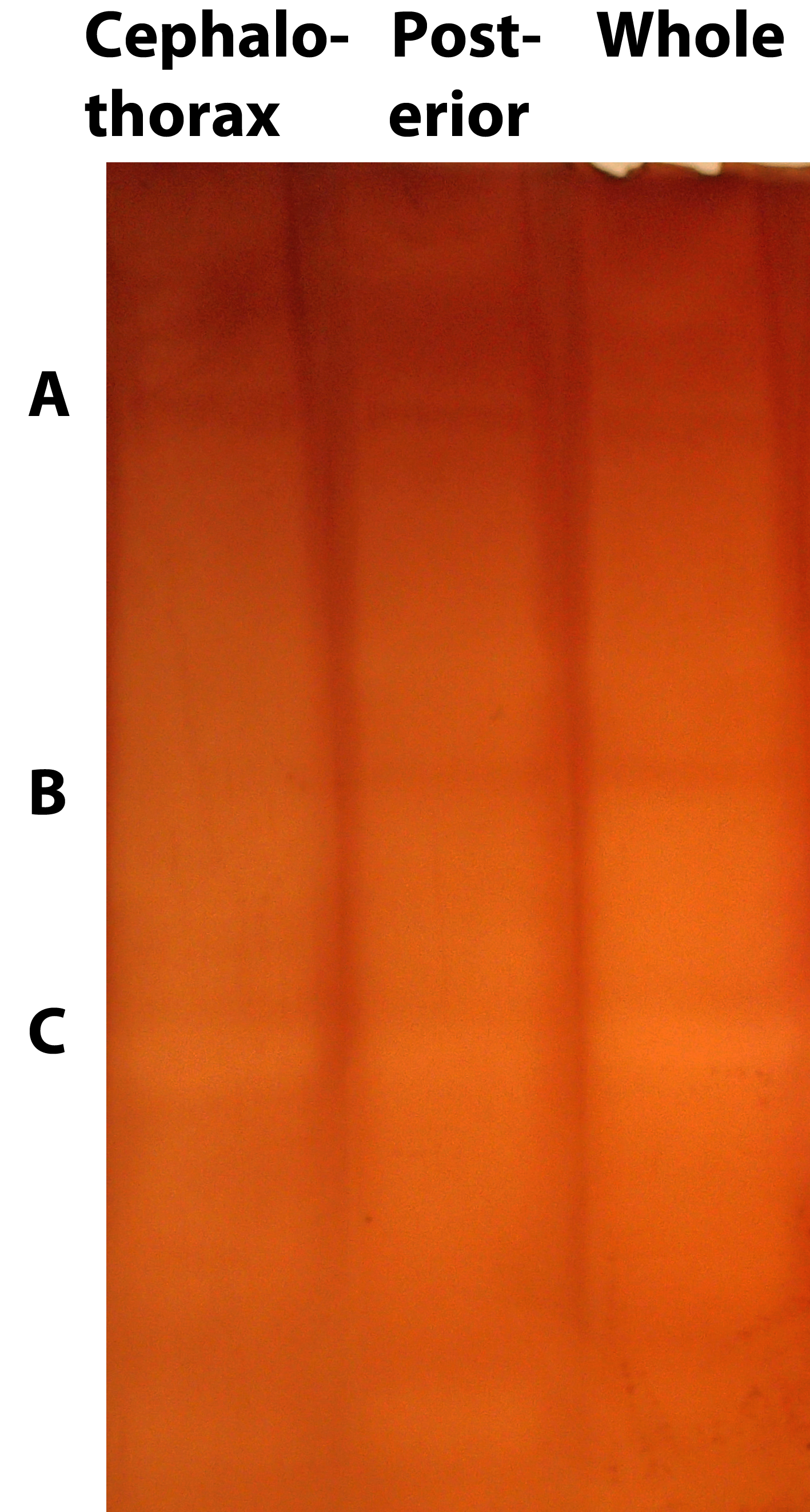

Supplement: S3 Fig — Native polyacrylamide gel electrophoresis of Lepeophtheirus salmonis acetylcholinesterases from cephalothorax segment, posterior segment and whole body tissues of female adult lice. Protein samples (50 μg) were loaded on 8% polyacrylamide gel. After running, the gel was activity-stained to visualize AChE bands according to Lewis and Shuttle (29). (TIF) [file pone.0125362.s003.tif]

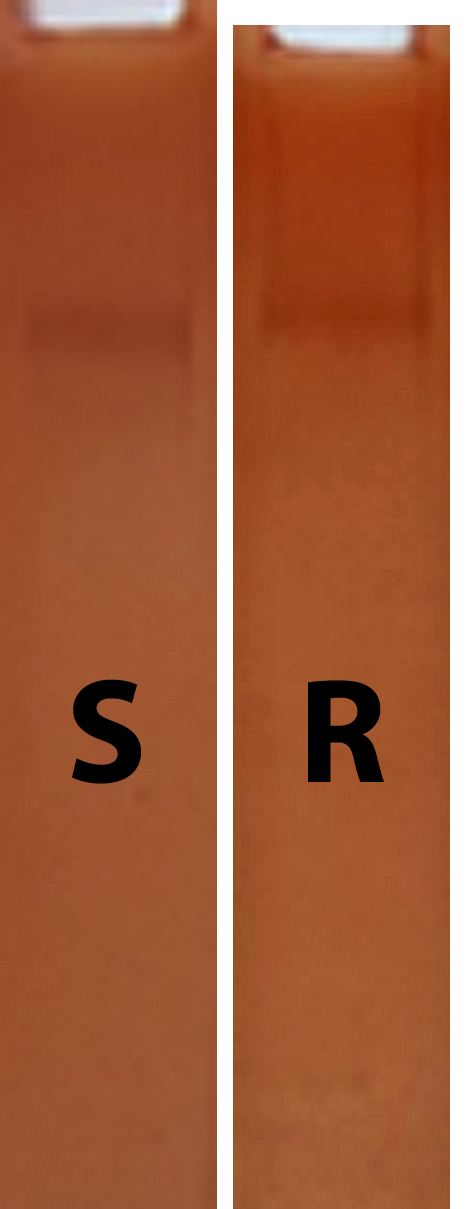

Supplement: S4 Fig — Native PAGE was performed on sensitive and resistant sea lice samples. Both the sensitive (lane S) and resistant (lane R) samples showed active AChE. Since preadult parasites were used for the experiment, which have a higher proportion of neural tissue compared to the body size, only one band was observed. Based on S3 Fig (MW and pI of AChE1a and AChE1b), this band possibly corresponds to AChE1a. (TIF) [file pone.0125362.s004.tif]
