# Supplementary material for: Identification and Molecular Characterization of Two Acetylcholinesterases from the Salmon Louse, Lepeophtheirus salmonis
Source: PLoS One. 2015 May 4;10(5):e0125362. doi: 10.1371/journal.pone.0125362 (PMC4418574; doi:10.1371/journal.pone.0125362)
Supplement: S2 File — (DOCX) [file pone.0125362.s006.docx]

**Generation of polyclonal antibodies against *L. salmonis* AChE1a and AChE1b**

The polyclonal antibodies were produced by GenScript (GenScript USA Inc., Piscataway, NJ, USA) using the peptide sequences (14 amino acid long peptide starting from 130-144 amino acid position in both the proteins) followed by immunization of rabbits. Serum specific antibodies were affinity purified on columns using immobilized antigen peptides.

**Western blot analysis**

After electrophoresis, the separated proteins were transferred to Hybond-N nitrocellulose membranes. Electrotransfer was performed at room temperature for 1 hour at 100V. After transfer, the membranes were blocked with 5% skimmed milk in PBS buffer containing 0.1% Tween-20 (PBST) for 1 hour at room temperature with gentle shaking. The membranes were then incubated with anti-AChE1a and anti-AChE1b polyclonal antibodies (custom prepared by GenScript USA Inc, Piscataway, NJ, USA), diluted in PBST buffer at 1:200, overnight at 4^o^C. After three washings with PBST buffer, the membranes were incubated with a biotin-labeled goat anti-rabbit IgG secondary (Invitrogen, CA, USA) antibody diluted 1: 3000 in PBST for 1 hour. After three washings the blots were incubated in streptavidin – biotinylated alkaline phosphate solution (Bio-Rad, CA, USA) for 1 hour at room temperature. The detection of antigen-antibody complexes on the bands was visualized using alkaline phosphate conjugate substrate kit (Bio-Rad, CA, USA) according to manufacturer’s protocol.
